# The pathogenesis and potential therapeutic targets in sepsis

**DOI:** 10.1002/mco2.418

**Published:** 2023-11-20

**Authors:** Wendan Zhang, Honghong Jiang, Gaosong Wu, Pengli Huang, Haonan Wang, Huazhasng An, Sanhong Liu, Weidong Zhang

**Affiliations:** ^1^ Shanghai Frontiers Science Center of TCM Chemical Biology Institute of Interdisciplinary Integrative Medicine Research Shanghai University of Traditional Chinese Medicine Shanghai China; ^2^ Faculty of Pediatrics National Engineering Laboratory for Birth defects prevention and control of key technology Beijing Key Laboratory of Pediatric Organ Failure the Chinese PLA General Hospital Beijing China; ^3^ Shandong Provincial Key Laboratory for Rheumatic Disease and Translational Medicine The First Affiliated Hospital of Shandong First Medical University & Shandong Provincial Qianfoshan Hospital Jinan Shandong China; ^4^ Department of Phytochemistry School of Pharmacy Second Military Medical University Shanghai China; ^5^ The Research Center for Traditional Chinese Medicine Shanghai Institute of Infectious Diseases and Biosecurity Shanghai University of Traditional Chinese Medicine Shanghai China; ^6^ Institute of Medicinal Plant Development Chinese Academy of Medical Sciences and Peking Union Medical College Beijing China

**Keywords:** molecular mechanism, pathogenesis, sepsis, signaling pathways, therapeutic drugs

## Abstract

Sepsis is defined as “a life‐threatening organ dysfunction caused by dysregulated host systemic inflammatory and immune response to infection.” At present, sepsis continues to pose a grave healthcare concern worldwide. Despite the use of supportive measures in treating traditional sepsis, such as intravenous fluids, vasoactive substances, and oxygen plus antibiotics to eradicate harmful pathogens, there is an ongoing increase in both the morbidity and mortality associated with sepsis during clinical interventions. Therefore, it is urgent to design specific pharmacologic agents for the treatment of sepsis and convert them into a novel targeted treatment strategy. Herein, we provide an overview of the molecular mechanisms that may be involved in sepsis, such as the inflammatory response, immune dysfunction, complement deactivation, mitochondrial damage, and endoplasmic reticulum stress. Additionally, we highlight important targets involved in sepsis‐related regulatory mechanisms, including GSDMD, HMGB1, STING, and SQSTM1, among others. We summarize the latest advancements in potential therapeutic drugs that specifically target these signaling pathways and paramount targets, covering both preclinical studies and clinical trials. In addition, this review provides a detailed description of the crosstalk and function between signaling pathways and vital targets, which provides more opportunities for the clinical development of new treatments for sepsis.

## Graphical abstract

In this review, we describe the possible pathogenesis of sepsis, summarize the main regulatory pathways that intervene in sepsis and the main regulatory target proteins of sepsis, and the recent exploration of the main intervention molecules in sepsis.

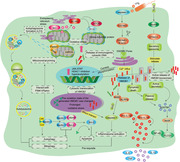

## 1 INTRODUCTION

Throughout history, the term “sepiton” was utilized by Hippocrates to express the possibility of harmful biological decay transpiring within the body.^1^, ^2^ In earlier periods, sepsis was defined as a syndrome of systemic inflammation in reaction to microbial infection. This syndrome is characterized by extensive tissue damage and systemic inflammation.^3^ However, recent advancements in understanding the pathophysiology of sepsis have unveiled its true nature, indicating that the definition of sepsis cannot solely rely on it being an inflammatory syndrome.^4^ Clearly, the concept of systemic inflammatory response syndrome (SIRS) is too broadly employed in critically ill patients, failing to encompass the clinical heterogeneity and dynamics of real‐life scenarios. In 2016, international guidelines were revised to introduce the concept of sepsis 3.0. Sepsis 3.0 defines sepsis as life‐threatening organ dysfunction caused by an impaired host response to various infections, whether bacterial, fungal, protozoan, or viral, which includes the recent severe acute respiratory syndrome coronavirus 2 responsible for coronavirus disease 2019 (COVID‐19).^5^ Sepsis is a multifaceted syndrome that manifests in different ways as the host's regulation is disrupted in response to infection. Traditionally, sepsis has been mainly attributed to the host's overly inflammatory immune response to infection. Consequently, most clinical studies in the 1990s focused on therapies aimed at limiting excessive inflammation, without any substantial success.^6^, ^7^

The morbidity and mortality of sepsis are tremendous, and the continuous growth of this condition has caused a substantial burden on global healthcare.^8^, ^9^ World Sepsis Day was created on September 13, 2012, with the main goal of raising public awareness about the severity of this perilous illness. Shockingly, in 2017 alone, approximately 48.9 million individuals across the globe were afflicted by sepsis, leading to a staggering 11.0 million deaths. These sepsis‐related fatalities accounted for nearly one‐fifth of all deaths worldwide. The immense scale and devastating impact of sepsis cannot be underestimated.^10^ Therefore, sepsis is a critical public health issue, causing considerable economic consequences around the world. The World Health Organization has called on member states to enhance their efforts in preventing, diagnosing, and managing sepsis.^11^, ^12^ This can only be accomplished by gaining a deeper understanding of the underlying mechanisms of sepsis and implementing more precise and efficient treatment strategies.^13^ Sepsis is a multifaceted condition that arises as a result of an unbalanced immune response to infection. It is characterized not only by an overwhelming inflammatory reaction but also by excessive suppression of the immune system. To effectively address sepsis, it is crucial to comprehend the complexities of sepsis development and the intricate interplay between inflammation and immunosuppression.^14^, ^15^ Despite significant advancements in medical technologies, particularly in the field of anti‐infective treatments, there is still no conclusive remedy to address the prevalent issue of sepsis. Furthermore, individuals afflicted with sepsis encounter enduring and severe complications encompassing physical, psychological, and cognitive impairments, even following therapeutic interventions.^16^ To address these issues, new criteria for sepsis recognition and intervention should focus more on regulating key targets in signaling pathways rather than determining signs of inflammation. Therefore, it is extremely urgent to develop prevention methods and effective treatment measures for sepsis to solve human health problems.

An improved understanding of sepsis has revealed that it is not just a systemic inflammatory response or immune disease process but also entails changes in the function of multiple organs in the body.^17^ Essentially, sepsis can be seen as a battle between the pathogen and the host immune system, where the outcome is determined by the delicate balance between pro‐ and anti‐inflammatory pathways. This imbalance can result in multiple organ dysfunction and ultimately decide the fate of the individual.^18^, ^19^ Significant advancements have been made in understanding how sepsis causes organ injury at the molecular, cellular, and organ levels. The pathogenesis of sepsis is an extremely complex and diverse pathophysiological process that leads to an imbalance in homeostasis at the molecular, cellular, and organ levels, eventually leading to organ dysfunction and even death.^13^, ^20^, ^21^, ^22^ Herein, a comprehensive review is presented concerning the dysregulated/altered pathways and molecules implicated in sepsis, along with a potential molecular mechanism for the development of sepsis. Additionally, detailed explanations were given regarding the interactions among these pathways. We also delineated novel candidate therapeutic targets and drugs that might contribute to the treatment of sepsis.

## 2 REGULATORY MECHANISMS INVOLVED IN SEPSIS

In the process of basic and clinical complementary and progressive research, many new mechanisms, targets, and therapeutic molecules of sepsis have been discovered and validated.^23^, ^24^, ^25^ The findings have attracted increasing attention, attempting to translate them into clinical outcomes. Expanding from the macro level to micro details, dissecting the essence of sepsis, transforming from a simple definition of septic shock in the past to a heterogeneous lesion with multiple organ dysfunction caused by infection.^26^, ^27^ Variations of this nature result in the inability to identify a specific cause in the clinical presentation of patients with sepsis and thus to implement interventions.^3^, ^28^ In the past, the clinical management of sepsis mainly took the form of the administration of interferon, vasopressors, or intravenous fluids. However, these interventions have some potential risks and safety issues, such as antibiotic resistance. Therefore, further evaluation is needed to determine the safety and long‐term impact of these applications on human health.^19^, ^22^, ^29^ With the continuous progress of science and technology and the rapid development of clinical medicine, the understanding of the development of sepsis has risen from the initial excessive inflammatory reaction leading to immunosuppression, mitochondrial dysfunction, endoplasmic reticulum (ER) emergencies, cell necrosis (pyroptosis, apoptosis, etc.), neural network damage, complement system disorder leading to coagulation abnormalities, and so on.^4^, ^30^ In this section, we review the main regulatory molecular mechanisms involved in the occurrence and development of sepsis and emphasize potential new candidate therapeutic targets and drugs for treating sepsis.

### 2.1 Inflammation and immune

The improper presence of microorganisms (bacteria, etc.) and their products can trigger a host immune response that is critical for maintaining and restoring homeostasis but can lead to tissue damage if excessive.^31^ In practice, the greater the inflammatory response is, the stronger the cellular damage and thus the higher the risk of organ dysfunction.^18^ At its onset, sepsis manifests as an overwhelming release of inflammatory mediators (cytokine storms) in response to infection.^32^, ^33^ The immune response to infection can work with innate immunization defense, components of the innate immune system at the site of pathogen exposure that activate and recruit circulating immune cells.^16^, ^34^ Immune cells have pathogen‐recognition receptors (PRRs) on their surface that are activated by binding to pathogen‐associated molecular patterns (PAMPs) or damage‐associated molecular patterns (DAMPs), such as bacteria, burn, trauma, and so on. Receptor binding triggers intracellular signaling cascades, which in turn leads to the production of associated inflammatory mediators, thus initiating an immunoinflammatory cascade.^35^, ^36^ The ensuing chain reaction will eventually lead to the activation of an even stronger “armed force,” which is the adaptive immune response.^37^ This explosive activation and the resulting immune “cytokine storm” have been identified as the pathogenic pathway for sepsis.^33^, ^38^

**FIGURE 1:**
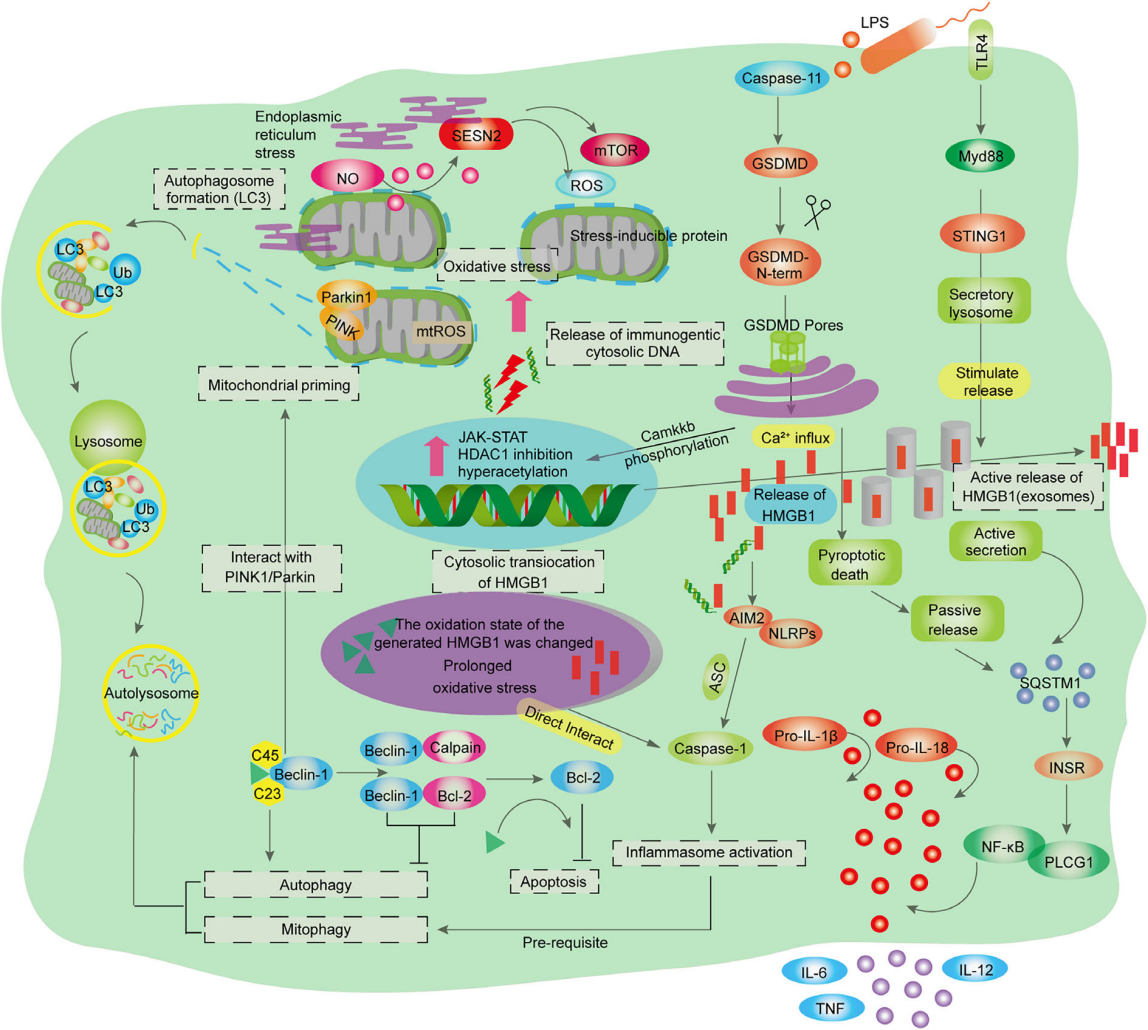
Altered innate immune function and cellular homeostasis after sepsis. In immune cells, the complementarity and regulation of inflammasomes, cell death pathways, and oxidative stress contribute greatly to the maintenance of host cell survival and homeostasis. MtROS and mtDNA activate the JAK–STAT pathway and trigger histone deacetylase 1 (HDAC1) inhibition, which is required for HMGB1 to play a regulatory role (hyperacetylation and cytosolic translocation). The massive release of all stimuli‐induced HMGB1 by exosomes is an inflammatory mediator that marks the prelude of sepsis. AIM2 or NLRPs and dsDNA commence the inflammasome, which in turn triggers caspase‐1‐driven responses. These responses act as prerequisites for the induction of autophagy/mitophagy through pathways mediated by beclin1. In addition, HMGB1 induces autophagy by releasing Beclin­1 from Bcl2 after binding with Beclin­1 and then removing hazardous oxidative stress stimuli. PINK1 promotes the recruitment of Parkin to mitochondria, and subsequently, beclin‐1 interacts with pink/Parkin to further initiate mitochondrial priming and autophagosome generation, which then interact with lysosomes to induce mitophagy. Autophagy and mitophagy are the basic lines of defense that protect the body from injuries and regulate innate immune responses. During sepsis, macrophages secrete or release SQSTM1 actively or passively in response to extracellular or intracellular LPS, respectively. The released SQSTM1 then functions as an inflammatory mediator by activating the NF‐κB pathway, which is dependent on the INSR, contributing to the inflammatory response. Therefore, failure to activate protective autophagy/mitophagy may lead to accelerated death in sepsis.

**FIGURE 2:**
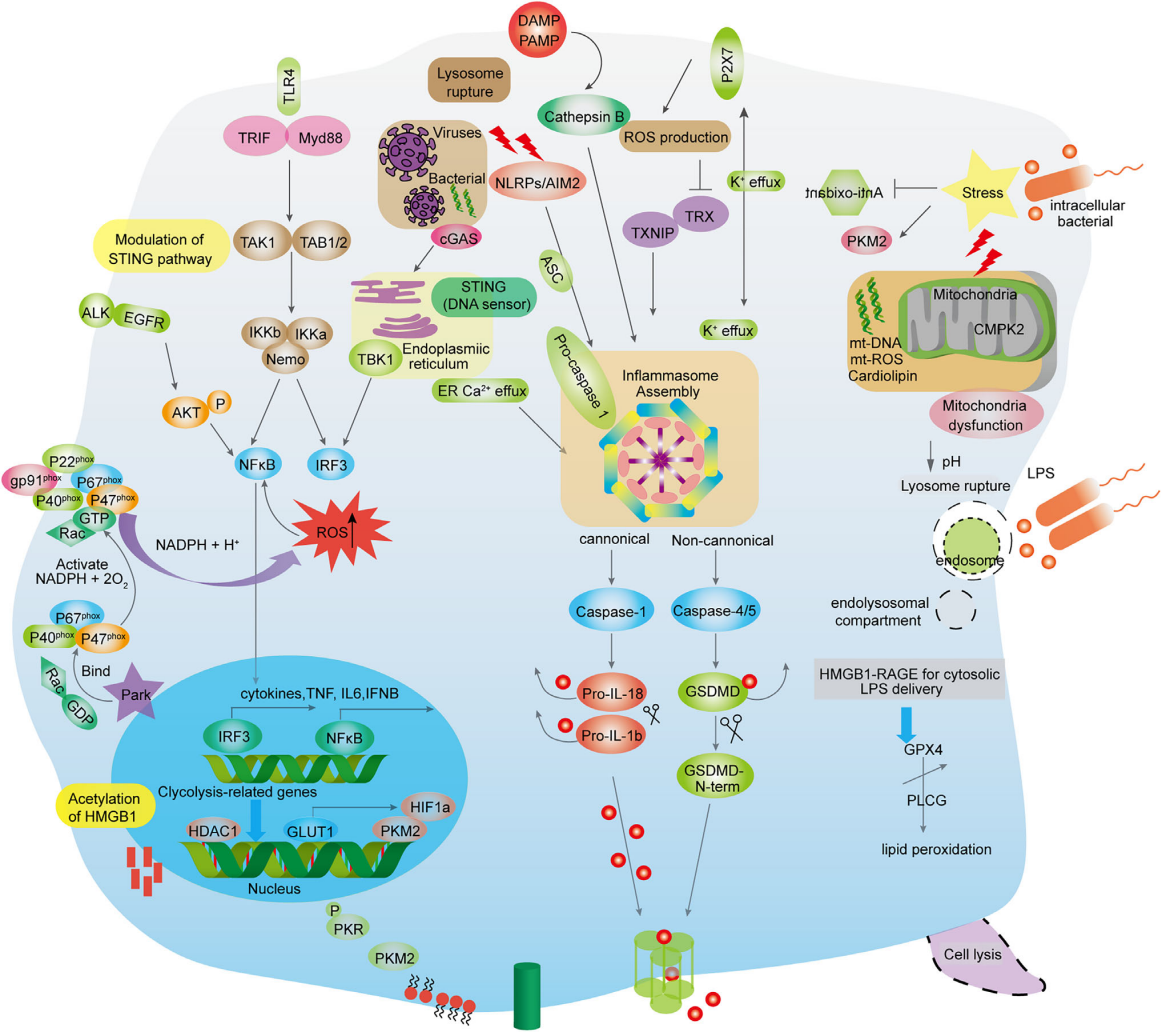
Aggregation of signaling pathways in innate immune cells. Inflammation is initiated at the initiation of sepsis by the sensing of PAMPs and DAMPs. Subsequently, multiple pathways activate and regulate innate immune responses through danger signals sensed by intracellular and membrane‐bound receptors. These signaling pathways toward IRF3 and NF‐κB initiate early phase inflammatory responses. In addition, LPS or HMGB1 provides an essential priming step of inflammasome activation. An activation step for AIM2/NLRP3 inflammasome assembly necessitates the presence of stimuli called PAMPs and DAMPs, which include crystals and ATP. This step leads to the cleavage of caspase, GSDMD, and pro‐IL‐1β/18, ultimately triggering canonical inflammasome activation and pyroptosis. Then, HMGB1 interacts with RAGE to deliver cytoplasmic LPS, which in turn triggers pyroptosis via a caspase‐11‐dependent pathway of noncanonical inflammasome pathway (equivalent to caspase‐4 and caspase‐5 in humans). AIM2 and cGAS‐STING detect cytosolic DNA and intracellular pathogens, triggering the assembly of the inflammasome and phosphorylation of IRF3. This activation leads to the induction of type I interferon (IFN) responses and inflammasome activation. In the context of sepsis, mitochondrial dysfunction, the generation of reactive oxygen species (ROS), and metabolic reprogramming further augment the production of HMGB1, a key proinflammatory cytokine. The inflammasome plays a pivotal role in the pathogenesis of sepsis, as it is intricately linked to stress signaling, activation of immune cells, and maintenance of cellular homeostasis. In the later stages of sepsis, the impaired process of macrophage activation can occur when the TLR/NF‐κB and/or TLR/MARKs signaling pathway is inactivated. This inactivation is induced by various stimuli such as LPS/LTA/PGN and proinflammatory cytokines. P47phox, a component of the NADPH oxidase proenzyme, interacts with Park 7, leading to the phosphorylation and translocation of P47Phox to the membrane. This translocation allows the formation of a holoenzyme complex. The subsequent activation of NADPH oxidase produces reactive oxygen species (ROS). These ROS molecules then trigger the downstream MAPK and NF‐κB signaling pathways that are involved in TLR signaling, ultimately resulting in macrophage activation. The activation of macrophages serves multiple functions, including the prevention of sepsis‐induced immunosuppression. This prevention is achieved through the release of proinflammatory cytokines, the elimination of pathogens, polarization into the M1 phenotype, and the enhancement of autophagy.

In the sepsis‐induced inflammatory response, both external factors derived from pathogens and internal factors released by damaged cells (such as lipopolysaccharide [LPS] and high‐mobility group box‐1 [HMGB‐1] protein) can recognize PRRs, including Toll‐like receptors (TLRs), C‐type lectin receptors (CLRs), retinoic acid inducible gene I (RIG‐I)‐like receptors, and NOD‐like receptors (NLRs), leading to the activation of downstream pathways.^39^ The activation of different receptors results in the translocation of transcription factors, such as nuclear factor‐κB (NF‐κB), into the nucleus and subsequent activation of target genes, which encode proinflammatory cytokines such as tumor necrosis factor (TNF), interleukin (IL)−6, IL‐12, and interferons (IFNs) (Figure 1).^40^ The secretion of proinflammatory factors progresses to a life‐threatening cytokine storm. Moreover, soluble cytosolic NLRs also contribute to the immune imbalance associated with sepsis.^41^, ^42^ The activation of specific NLRs is regulated by adaptor receptor‐interacting protein kinase 2 (RIP2) (also known as RICK), leading to NF‐κB activation and adaptor protein 1 activation. However, certain other NLRs (e.g., NLRP and NLRC4) participate in the assembly of distinct inflammasome protein complexes.^43^ The inflammasome cleaves procaspase‐1 into active caspase‐1, which then cleaves pro‐IL‐1β and pro‐IL‐18, resulting in the release of the highly inflammatory cytokines IL‐1β and IL‐18 and triggering a form of programmed cell death (known as pyroptosis) characterized by intense inflammation.^44^ When these mechanisms are driven by sepsis, they can become catastrophic and lead to a life‐threatening inflammatory cascade.^45^, ^46^ Dectin is one of the main CLRs that plays a significant role in various biological processes. This particular Dectin has the ability to induce the production of reactive oxygen species (ROS) and activate inflammatory responses through the action of SRC and SYK kinases.^47^ Interestingly, in addition to the PPRs mentioned above, some double‐stranded RNA receptors have been found, mainly including RIG‐I, melanoma differentiation‐associated gene 5, and laboratory of genetics and physiology 2, and are also associated with sepsis induced immune dysfunction.^48^, ^49^, ^50^ It has been noted that PRR can be activated by both exogenous PAMPs and endogenous DAMPs.^36^, ^51^ In relation to the induction of sepsis from within the body, it has been found that hepatocytes release a significant amount of HMGB‐1, which binds to bacterial endotoxin known as LPS (Figure 2).^52^, ^53^ This bacterial endotoxin is then transported to the cytoplasm through receptors called receptor for advanced glycation end products (RAGE), which are expressed in vascular endothelial cells (ECs) and macrophages.^54^ As a result of this process, caspase‐11‐mediated cell death, or apoptosis, occurs, ultimately leading to sepsis, multiple organ failure, and death.^55^, ^56^

### 2.2 Inflammation and coagulopathy

**FIGURE 3:**
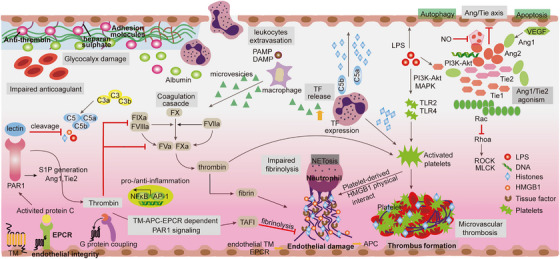
Activation of coagulation, complement, and endothelial cells during sepsis. The loss of antithrombogenicity and exposure of adhesion molecules are outcomes caused by the endothelial glycocalyx. These outcomes enable leukocyte adhesion, platelet recruitment, and the formation of a thrombus. Increased vascular permeability initiates the extravasation of leukocytes, the leakage of plasma proteins, and tissue edema. In response to immunogenic stimulation, macrophages and neutrophils express procoagulation tissue factors in the form of microvesicles that regulate coagulation cascades and prothrombin cleavage. The formation of FXa, thrombin, and fibrin occurs as a consequence of the activation of the cascade of proteolytic reactions. Fibroblasts, pericytes and epithelial cells constitutively express tissue factors that protect hemostasis and vascular integrity. During sepsis, NETosis interacts with platelet‐mediated responses to modulate innate immune responses and bacterial clearance. The occurrence of sepsis involves vessel injury induced by inflammation, thereby exposing the blood coagulation factors and leading to the formation of blood clots. The production of APC is mediated by a combination of thrombin and thrombomodulin (TM) and amplified by endothelial protein C receptor (EPCR). During sepsis, APC deactivates the coagulation cofactors FVa and FVIla, thus impeding the coagulation process. The recruitment of activated platelets and RBCs leads to thrombocytopenia and the presence of platelet‐rich thrombus. In addition, HMGBI and efDNA behave as procoagulants, expediting the formation of blood clots. The APC–EPCR interaction also allows for the conversion of PAR‐1 signaling into an anti‐inflammatory mode through ANG/TIE axis mediated activity and enhances endothelial integrity. In addition, TM/thrombin directly mediates immune‐coagulation, and ANG/TIE axis dysregulation is associated with decreased vascular stability during severe infection. Finally, disorders of coagulation, complement and endothelial function lead to microcirculation dysfunction and multiple organ damage, which are closely related to the extensive formation of inflammatory thrombi in sepsis.

Disseminated intravascular coagulation (DIC) is a life‐threatening syndrome that is commonly considered an organ dysfunction that is often present in sepsis, with severity positively correlated with mortality.^57^, ^58^ DIC overactivation is characterized by an intravenous coagulation cascade, exhaustion of anticoagulants, and inhibition of fibrinolysis.^59^ The dysregulation of inflammation has been found to trigger the coagulation response in sepsis, while the activation of the coagulation reaction exacerbates the inflammatory response.^29^ This highlights the crucial interaction between inflammation and coagulation, which is seen as the primary driver of sepsis pathogenesis.^60^ Throughout the onset and progression of sepsis, the generation of cytokines not only facilitates the activation of coagulation factors and platelets but also initiates the mechanism of anticoagulation (such as the system of antithrombin, the system of activated protein C (APC), and the inhibitor of tissue factor [TF] pathway). This can result in the leakage of blood vessels, DIC, and extensive accumulation of fibrin in either the blood vessels or tissues (Figure 3).^61^, ^62^ In general, coagulation activation is controlled by three vital physiological anticoagulant pathway systems, including the TF pathway inhibitor system (TFPI), APC system, and antithrombotic system, which complement each other to modulate coagulation activation. In the early stages of sepsis, insufficient balance of TFPI in TF‐dependent coagulation events supports impaired physiological functions of anticoagulants identified during sepsis.^63^ In addition, hyperinflammation and innate immunity work together in coordination to amplify the coagulation cascade, in which bacterial‐derived products and DAMPs (such as neutrophil extracellular traps [NETs], HMGB1 and cell‐free DNA [cfDNAs]) contribute to the development of DIC through multiple pathways.^25^, ^64^ In the advanced phases of sepsis, immune cell apoptosis (such as dendritic cells [DCs] and lymphocytes) results in immune stagnation by diminishing the host's capacity to eliminate intruding microorganisms and enhancing susceptibility to subsequent infections.^65^, ^66^ DIC, a key factor in multiple organ failure during sepsis, manifests as the formation of numerous microvascular blood clots, subsequently depleting platelets and coagulation factors, ultimately resulting in impaired hemostasis.^67^ In contrast, these thrombin and coagulation factors exhibit proinflammatory activity by cleaving protease‐activated receptors (PARs) to form a vicious cycle in which increased inflammation and coagulation further enhance the mortality rate of sepsis.^25^, ^57^, ^68^

### 2.3 Complement system

In the late 19th century, scientists made an interesting discovery—a plasma element known as the complement system. This component, which was found to be thermally unstable, served as a complement to antibodies in the process of eliminating microorganisms. This is why it was named “complement.”^69^, ^70^ However, it is important to recognize that the complement system can be both beneficial and detrimental. When dysregulated or excessively activated, it can turn against its host, causing harm to tissues, resulting in organ failure, and, in severe cases, leading to mortality.^71^, ^72^ The function of complement is beyond infectious defense, which promotes tissue regeneration and repair, facilitates organ development, and can be transformed and coordinated with plentiful processes, including hemostasis leading to inflammation that causes thrombosis.^71^ During the treatment phase of patients with sepsis, chemical reperfusion injuries (myocardial infarction, stroke, and transplant dysfunction) trigger (DAMPs) that trigger complement activation in chronic nervous system and rheumatic diseases, thereby contributing to inflammatory and immune disorders.^73^, ^74^ Dysregulation of the complement system marks a disruption in host recognition of defense signaling pathways associated with inflammation, coagulation, and bacterial cell lysis, and is a prelude to multiorgan dysfunction in sepsis.^75^

The activation of complement cascades is a widely acknowledged phenomenon that occurs via three distinct pathways. These pathways include the classical pathway (triggered by the complex formed by antigens and antibodies), the alternative pathway (initiated by factors B and D), and the lectin pathway (detected via the recognition of mannose derived from pathogens).^76^ After the complement system was activated, these three pathways gathered at the C3 level to produce cleavage products C3a and C5a, as well as the terminal membrane‐attacking complex C5b, which forms pores in the cell membranes of bacteria and cells, eventually leading to lysis (Figure 3).^77^ C3a, C5a, and C5b mediate antibacterial responses and proinflammatory effects by interacting with cell membrane receptors and triggering crosstalk among multiple signaling pathways. In sepsis, the innate immune functions of neutrophils are impaired due to excessive C5a generation, for example, chemotaxis, phagocytosis, and H_2_O_2_ production are blocked.^78^, ^79^ Thus, complement‐mediated activation of neutrophils is not only responsible for diverse significant impacts in sepsis but also assumes a contradictory function in innate defense.

Furthermore, sepsis is characterized by an early excessive production of C5a, which leads to an unregulated inflammatory response, ultimately resulting in tissue damage and the failure of multiple organs.^80^ Sustained complement activation can cause a massive release of C5a, which may impair the innate immune response of neutrophils, resulting in reduced inflammation and impaired ability to kill bacteria.^81^, ^82^ Moreover, elevated levels of C5a can impede the targeted migration of neutrophils. Notably, the secretion of TNF by neutrophils can inhibit the transcription of NF‐κB, as induced by the C5a‐mediated increase in IκBα levels. Furthermore, C5a disrupts the C3a–C3aR axis and promotes the premature expulsion of granulocytes and hematopoietic stem cells from the bone marrow, leading to a less targeted but more progressive inflammatory response.^83^ Through C5a signaling, CXCR4 levels on granulocytes are concurrently reduced, while proteases are released, resulting in the degradation of matrix proteins and inhibition of the effect of stromal cell‐derived factor 1, which causes phenotypic changes in neutrophils.^84^ Moreover, activation of the complement system triggers the cross‐activation of the NOD‐like receptor protein 3 (NLRP3) inflammasome and prethrombotic pathways (Figure 2).^85^ Pentraxins (such as CRP, SAP, PTX3) released in response can initiate the classical pathway by interacting with C1q.^86^ Apart from pentraxins, the complement system can be directly activated by coagulation proteases through the extrinsic protease pathway (Figure 2).^87^ The uncontrolled activation of the complement system during sepsis contributes to tissue damage and dysfunction in organs.

### 2.4 Mitochondrial dysfunction

Mitochondria play a vital role in energy generation, protein synthesis, regulation of cell growth and cycle, and catabolism.^88^, ^89^ Nevertheless, in cases of sepsis, the mitochondrial damage or malfunction can disrupt cellular metabolism,^41^, ^90^, ^91^, ^92^ impede energy production, and trigger oxidative stress.^93^, ^94^ Consequently, this may provoke apoptosis in both organ and immune cells, ultimately leading to immune disorders, multiple organ failure, and potentially fatal outcomes.^95^, ^96^ It has been proven that the involvement of specific NLRP3 inflammasome activators could expand mitochondrial instability, resulting in pyroptotic cell death and enhancing the formation of pores in the plasma membrane, which then release inflammatory factors through caspase‐1 dependent mechanisms. However, excessive ROS due to electron transport chain damage, Ca^2+^ overload, or reduced endogenous antioxidants can also trigger patterns of cell death, such as apoptosis and autophagy.^97^, ^98^, ^99^ In the pathogenesis of sepsis, mitochondrial function is disorganized, mainly manifested by a decrease in oxidative phosphorylation, an increase in ROS production, the effects of hormonal changes, downregulation of the genes encoding mitochondrial proteins and an increase in apoptosis, which eventually leads to altered mitochondrial biogenesis.^100^, ^101^, ^102^ Damaged mitochondria release a cascade of DAMPs, further activating and enhancing the immune response.^103^, ^104^ The changes in mitochondrial function that occur early in sepsis are an adaptive mechanism by which mitochondria protect cells. For example, the production of harmful ROS is regulated by oxidative phosphorylation.^105^, ^106^ In addition, in the early stages of sepsis, mitochondrial autophagy and mitochondrial biogenesis are increased to limit mitochondrial dysfunction.^107^, ^108^ Therefore, the development of sepsis is accompanied by changes in different mitochondrial functions.

Furthermore, molecular events resulting from mitochondrial dysfunction in sepsis trigger the activation of caspase‐1, which intensifies the response to inflammatory responses, such as perturbations of membrane permeability and damage to the mitochondrial network.^109^, ^110^ Although serum proinflammatory factors and antiapoptotic proteins have been shown to play important roles in the activation and inhibition of the NLRP3 inflammasome, the pathogenesis of sepsis‐induced acute respiratory distress syndrome is primarily an elevated intracellular oxidative stress‐mediated apoptotic event.^111^, ^112^, ^113^ The alterations in the stage of organ function and how persistent organ dysfunction further affects mitochondrial performance remain puzzling, but the confusion is still the subject of translational medicine research because these periodic responses illustrate the potential of targeted therapy to alleviate the organ dysfunction induced by sepsis.

### 2.5 Pyroptosis

Pyroptosis is a lytic type of programmed necrosis that is linked to the secretion of proinflammatory cytokines. This process has pivotal functions in facilitating the defensive response of the innate immune system against microbial infections and invading pathogens.^114^, ^115^, ^116^, ^117^ These signature genes in pyroptosis are NLRP3, apoptotic speck‐like protein containing caspase activation and recruitment domains (CARD) (ASC), cleaved Caspase‐1, Gasdermin‐D (GSDMD) p30, IL‐1β, and IL‐18, which play a prominent role in the response to infection, ultimately fueling inflammation (Figure 2).^21^, ^109^, ^110^, ^118^, ^119^ The expression levels of inflammatory genes show complex correlations to a certain extent, ultimately leading to the rampant proliferation of inflammatory gene profiles (such as NLRP3, NLRC4, NOD, IL‐1β, and IL‐18), while in sepsis studies, the same gene regulation pattern was observed with a higher amplitude of change, revealing its clinical correlation with the severity of sepsis.^39^, ^120^, ^121^ It has been established that NLRP3 inflammasome activation requires “prime” and “activation.”^122^, ^123^ The NF‐κB signaling pathway is an essential priming event that ensures accurate response of the inflammasome to LPS stimulation and prevents inappropriate activation of NLRP3.^124^, ^125^, ^126^ Recent research has suggested that the initiation of NLRP3 deubiquitination may occur through a nontranscriptional mechanism. Specifically, these studies have proposed that mitochondrial ROS are responsible for inducing priming by causing the deubiquitination of the NLRP3 inflammasome. This finding challenges the traditional belief that NLRP3 deubiquitination is primarily regulated by transcriptional processes. Instead, it suggests that a direct interaction between mitochondrial ROS and the NLRP3 inflammasome is involved in this important regulatory step. Further investigation is needed to fully understand the exact molecular mechanisms underlying this nontranscriptional initiation of NLRP3 deubiquitination.^126^, ^127^, ^128^ In addition, LPS‐induced cytidine/uridine monophosphate kinase 2 (CMPK2), which is rate limiting for mtDNA synthesis, reduced NLRP3 inflammasome assembly (Figure 2).^129^, ^130^ In the absence of NLRP3 activators, prime‐signaling provided by NF‐κB or CMPK2 is insufficient for activating inflammasomes, suggesting that NLRP3 inflammasome activators play a pivotal role in triggering inflammation.^131^, ^132^, ^133^ NLRP3 inflammasome activators include several physically and chemically diverse extracellular stimuli, such as extracellular adenosine triphosphate (ATP) or uric acid crystals, exogenous particulates such as aluminum salts and titanium dioxide, pathogens and pore‐forming toxins.^134^, ^135^, ^136^, ^137^, ^138^ During sepsis, ATP activates the P2 × 7 receptor and NLRP3 signal transduction in an autocrine manner after activation of the inflammasome, leading to changes in metabolic status and convergence of inflammasome signals and is associated with increased lethality.^139^, ^140^ Mechanistically, these inflammasome activators function to increase downstream binding of NEK7 with NLRP3^141^, ^142^, ^143^ and regulate NLRP3 oligomerization, inflammasome assembly, and catalytic cleavage of procaspases.

Recently, a novel TLR4‐independent mechanism for triggering pyroptosis was discovered.^144^, ^145^ Intracellular LPS directly binds to the procaspase‐11 recruitment CARD domain, which then oligomerizes and activates procaspase‐11.^146^, ^147^ The induction of pyroptosis, which is a critical event in septic mice, is dependent on Caspase‐11 activity.^44^, ^148^, ^149^, ^150^ Additionally, pyroptosis in human mononuclear cell lines is mediated by homologous caspase‐4/5. The activation of NLRP3 is regulated by Caspase‐11, and it is important to note that the cleavage of GSDMD by active caspase‐1/4/5/11 leads to the release of the functional gasdermin‐N domain. This released domain subsequently forms pores in the membrane, facilitating the active release of inflammatory cytokines and intracellular components (Figure 2).^151^, ^152^, ^153^, ^154^, ^155^, ^156^ The ability of the gasdermin family to form pores plays a significant role in the unique molecular and structural mechanisms underlying pyroptosis.^157^, ^158^ Interestingly, the pore formed by GSDMD‐N enables nonselective ion diffusion without increasing osmotic pressure, unlike membrane blebbing and cell swelling.^159^, ^160^ Mitochondrial dysfunction may trigger the release of oxidized mtDNA and cardiolipin into the cytosol, consequently activating the NLRP3 inflammasome and leading to the production of IL‐1β. Furthermore, the activation of inflammasomes can be driven by the significant efflux of potassium induced by toxins such as α‐toxin, which form potassium channels.^161^, ^162^, ^163^, ^164^

### 2.6 Neuroinflammatory reflex

Homeostasis of neuroinflammatory reflex system interactions is also considered to be a crucial part of the host response during sepsis.^165^ The neuroinflammatory response to sepsis is not due to infection of the brain but rather systemic inflammation associated with innate immunity to the central nervous system (CNS) through various pathways.^166^ LPS can activate TLR2 and TLR4 surrounding the cerebrum, choroid plexus and leptomeninges, thus profoundly inducing the transcription of inflammatory mediators throughout the brain parenchyma.^167^ However, the neuroinflammatory reflex immune system can suppress inflammation by activating the splenic nerve in the celiac nerve plexus, which causes the spleen to release norepinephrine (NA) and CD4^+^ T cell subsets to secrete acetylcholine. The characteristics of cerebral dysfunction in sepsis are altered mental states, including disorientation and confusion.^168^, ^169^ Patients with severe formal sepsis related to cerebral dysfunction may develop coma.^170^, ^171^ Importantly, sepsis‐associated cerebral dysfunction can be present without other organ dysfunctions (such as liver failure) that might be associated with encephalopathy.^40^ As a result, patients experiencing cerebral dysfunction associated with sepsis face a significant mortality risk and commonly endure prolonged cognitive and functional aftereffects. The activation/dysfunction of the vagus nerve and endothelium triggers the release of cytokines and neurotoxic mediators. This mechanism exemplifies the brain's immune system operations by transmitting distinct signals along various pathways to the brain. These incoming signals subsequently prompt the CNS's outgoing response, activating both the sympathetic and parasympathetic branches of the autonomic nervous system. Consequently, the activation of the adrenal axis is regulated, which counteracts the inflammatory process and restores cardiovascular homeostasis.^172^, ^173^ Additionally, the production of cytokines is primarily regulated by a specific division of the autonomic nervous system, particularly the sympathetic branch.^174^ Existing data suggest that NA can react to LPS, and elevated levels of NA were observed in close proximity to immune cells, inhibiting the expression of TNF‐α and IL‐12 (proinflammatory cytokines), while promoting the expression of IL‐10 (an anti‐inflammatory cytokine).^175^ Furthermore, acetylcholine signaling, as a neurotransmitter, also plays a crucial role in regulating the inflammatory response of sepsis, thereby introducing novel avenues for sepsis treatment.^176^, ^177^

### 2.7 Necroptosis

In contrast to caspase‐dependent programmed cell death, necroptosis is an unprogrammed and autonomous form of cell death that can be activated by various virulence factors discharged by the pathogen. However, sepsis‐induced changes in organ perfusion and hypovolemia can also contribute to necrosis.^178^ This process, in turn, can bolster the release of extracellular “alarm proteins” such as HMGB1, thereby exacerbating local inflammation. Therefore, necroptosis plays an essential role in cell death during sepsis. Additionally, necroptosis triggers inflammation and immunogenic events in different diseases by interacting with death receptors and diverse cytoplasmic protein kinases. Unlike necrosis, which usually results in irreversible pathological damage, necroptosis is employed by organisms to acquire appropriate levels of energy supply and promote cell regeneration.^179^, ^180^ Initially, defined as a type of TNF‐induced necrosis, necroptosis was subsequently recognized as a specific kinase‐dependent process after being effectively subdued by RIPK1 inhibitors.^181^, ^182^, ^183^ Necroptosis is specifically regulated by signal transduction proteins known as RIPK1 and RIPK3, which operate in complexes of membrane‐associated proteins. These complexes are activated by TLR (TLR3, TLR4) via rhomboid‐like (RHIM) domain‐containing proteins (TRIF, DAI).^184^, ^185^, ^186^ Successive stimuli induce interactions between coupling proteins and kinases and then initiate necroptosis cytoplasmic complexes, thereby responding by inducing signal transduction associated with cell death/survival outcomes.^187^, ^188^, ^189^ Necroptosis can be induced by various stimuli other than TNF/TNFR1, including immune death signals (FASL/TRAIL), bacterial and cellular stress signals (LPS/TLR4, poly(I:C)/TLR3), and type I/II IFNs.^190^, ^191^, ^192^ These stimuli trigger the formation of necrosomes by activating death receptors. Furthermore, the recruitment of RIPK1 to FAS without an inhibitor of apoptosis leads to the assembly of necrosomes and necroptosis.^193^ On the other hand, activation of the cytosolic complex promotes cell survival and proinflammatory signaling through NF‐κB and MAPK activation.^194^, ^195^ However, the mechanisms that determine whether a cell undergoes apoptosis, necroptosis, or cell survival are still not fully understood. Genetic studies have shown that deficiencies in FADD or Caspase‐8 can result in RIPK3‐mediated necroptosis, leading to embryonic lethality and inflammation.^196^, ^197^, ^198^ In clinical sepsis, levels of RIPK3 were found to be significantly elevated at different time points.^199^, ^200^ Moreover, higher levels of RIPK3 were associated with an increased incidence of organ dysfunction and septic shock, highlighting the pathological effects of RIPK3‐mediated pathways.

Collaboration between necroptosis and pyroptosis can expand the transmission of inflammatory signals, cause tissue injury, and exacerbate organ failure, both of which play pivotal roles in the progression of sepsis.^201^, ^202^ Although both processes can be triggered by similar stimuli, different pathways are involved in intracellular signaling, targeting distinct death regulatory proteins, specifically RIPK3 or GSDMD.^203^, ^204^, ^205^ The release of intracellular components differs in the active stage of sepsis development; necroptosis leads to the release of HMGB1, while pyroptosis results in the release of the proinflammatory cytokines IL‐1β and IL‐18.^26^, ^206^ To explore potential synergistic effects in sepsis‐induced injury, studies have investigated the protective effects of dual deletions of RIPK3 and GSDMD. Knockout (KO) of RIPK3/GSDMD or mixed lineage kinase domain‐like/GSDMD has demonstrated protective effects against septic shock, systemic coagulation, and multiple organ injury caused by necroptosis and pyroptosis double obstruction.^207^, ^208^ Both RIPK3 and GSDMD contribute to lytic cell death, which collaborates to amplify necrotic inflammation and the release of TF in macrophages and ECs, resulting in significant tissue damage (Figure 3). Hence, RIPK inhibition is proposed to play a protective role in the sepsis model. A recent in vivo study using LPS‐stimulated human kidney tubular epithelial cells showed increased RIPK3 expression, with enhanced insights into the unique role of RIPK3 in regulating mitochondrial function during sepsis, as evidenced by observations of mitochondrial depolarization in vitro.

### 2.8 Endothelial barrier dysfunction

The endothelium forms the inner layer of cells from blood vessels and lymphatics and plays a vital role in controlling blood flow and vascular tone as well as participating in immune responses.^209^ The destruction of the endothelial barrier is the most prominent phenomenon in the pathogenesis of sepsis. The integrity of the endothelial barrier is maintained by intracellular junction molecules, which can regulate the anticoagulant and anti‐inflammatory immune properties of the endothelial intima in the process of sepsis.^210^ Oxidative stress and inflammation lead to decreased vascular permeability, hypotension, and inadequate organ perfusion and eventually result in organ failure.^68^ These signature features play a key role in the pathogenesis of sepsis‐induced multiple organ failure.

Altered endothelial function was activated by disruption of intracellular junction molecule expression and dynamics of cytoskeleton contraction.^17^, ^211^, ^212^ In sepsis, a large number of novel therapeutic targets have been discovered, such as sphingosine 1‐phosphate 1, PAR1, vascular endothelial growth factor (VEGF), and angiopoietin 1 (Ang‐1), which have been found to play a regulatory role in endothelial dysfunction (Figure 3).^213^, ^214^, ^215^ The anti‐inflammatory properties of Ang‐1 derive from its ability to reduce leukocyte transmigration and cytokine production via Tie receptor 2 (Tie2) receptor signaling at the endothelial barrier.^47^, ^216^, ^217^ Inflammation‐induced release of Ang‐2 disrupts constitutive Ang1–Tie2 signaling by preventing Ang‐1 from binding to its receptor in sepsis, thereby reducing physiologic efforts to suppress ongoing inflammation.^218^, ^219^, ^220^ Therefore, the Ang/Tie2 pathway has broad impacts on vascular remodeling, inflammation, and EC survival.^221^, ^222^ In addition, Ang/Tie2 activation induces the expression of KLF2 via the phosphoinositide‐3 kinase/Akt pathway and counteracts VEGF‐mediated vascular permeability (Figure 3).^223^

### 2.9 ER stress

The ER is an intracellular organelle that is involved in protein translocation, folding, posttranslational modification, and further transport to the Golgi apparatus.^224^ Moreover, the ER provides a place for calcium storage, lipid synthesis and carbohydrate metabolism.^225^, ^226^ Under stress and inflammation conditions, the ER loses homeostasis in its function, which is termed ER stress. Unfolded or misfolded proteins accumulate in the ER during sepsis, altering its homeostasis and leading to oxidative stress and severe calcium disorders that result in ER stress.^227^, ^228^ Recent studies on ER stress signaling have revealed intriguing interactions between ER stress and sepsis‐related cell death.^229^, ^230^, ^231^ Recently, many studies have demonstrated that inhibition of ER stress can stabilize protein conformation, promote the transport of mutant proteins and improve ER folding ability.^232^ Therefore, ER stress is suggested as a potential therapeutic target for a variety of diseases, including diabetes, cystic fibrosis, sepsis and ischemic brain injury.^233^ In sepsis animal models, markers of increased ER stress (such as glucose‐regulated protein 94 [GRP94], CEBP homologous protein [CHOP], and caspase‐12) are detected in several organs, including the heart and liver, and these markers are directly connected with the extent of organ dysfunction, which may be a major cause of sepsis‐induced multiple organ failure.^234^, ^235^, ^236^ Studies have demonstrated that caspase‐12 deficiency confers protection in septic mice and that the presence of caspase‐12 leads to enhanced vulnerability to bacterial infection and septic mortality, suggesting a detrimental role of caspase‐12 in sepsis.^237^ These effects have been demonstrated in cecal ligation and puncture (CLP) models of sepsis, where ER stress leads to abnormal lymphocyte apoptosis in mice during sepsis, suggesting that the ER stress‐mediated apoptosis pathway may be a new target for the clinical prevention and treatment of sepsis‐induced lymphocyte apoptosis.^15^ During sepsis, the transcription factor CHOP is a major inducer of apoptosis in response to ER stress; however, recent evidence suggests an inflammatory role of CHOP as a mediator of the inflammatory response in sepsis.^238^ A major role for CHOP, which acts as an amplifier of the inflammatory response in the pathogenesis of sepsis, and the ability of H_2_S treatment to counter CHOP signaling via upregulation of NF‐E2‐related factor 2 are highlighted.^239^ ER stress causes abnormal apoptosis in septic animals, suggesting that ER stress‐mediated apoptosis represents a potential new target for the clinical prevention and treatment of sepsis.^240^, ^241^ However, these observations increase our knowledge of the biological mechanisms in the context of ER stress and sepsis and simultaneously shed light on new targets and suggest novel strategies for the treatment of this condition. Further research is warranted to elucidate the exact mechanism of how ER stress contributes to sepsis‐associated cell death.

### 2.10 Selective autophagy

Autophagy is a vital innate immune defense and self‐protective mechanism against microbial challenges. It is a self‐degradation process of basic metabolic mechanisms that degrades intracellular proteins and dysfunctional organelles to maintain cellular homeostasis and survival.^242^, ^243^ Increasing evidence shows that autophagy malfunction is associated with many human diseases, such as cancer, neurodegenerative diseases, liver diseases, and inflammatory diseases.^244^ It is noteworthy that autophagy can be mobilized in various organs in early sepsis, manifested by increased accumulation of autophagic vesicles and enhanced expression of autophagy‐related proteins.^245^, ^246^ Autophagy is likely to have a protective effect in sepsis through the mechanisms described below: elimination of pathogens, neutralization of microbial toxins, regulation of cytokine release, reduction in apoptosis, and promotion of antigen expression.^247^, ^248^, ^249^ Importantly, mitochondria‐produced ROS then oxidize HMGB1, which is released by apoptotic cells, resulting in the elimination of its immunogenicity and the inability to activate innate immune cells.^233^, ^250^, ^251^ However, during sepsis, the reduction of oxidized HMGB1 by thioredoxin is not efficient.^252^ HMGB1 directly interacts with the autophagy protein Beclin‐1, displacing Bcl‐2, which leads to the formation of autophagy initiation complexes and the elimination of harmful oxidative stress triggers.^243^, ^252^, ^253^, ^254^, ^255^ When binding to AIM2, all forms of intracellular HMGB1 that have been reduced by thiol display the highest affinity and subsequently initiate inflammasome signaling during redox stress (Figure 1).^256^ Therefore, activation of the inflammasome pathway is a critical prerequisite for promoting protective autophagy and enhancing cell survival.

Sepsis‐induced autophagy is initiated by binding PAMPs within the microbial structure to PRRs, such as TLRs, which further trigger different intracellular events and lead to increased autophagic activity by promoting the conversion of LC3‐I (microtubule‐associated protein 1A/1B‐light chain 3‐I) to LC3‐II.^257^, ^258^ The binding of LPS from gram‐negative pathogens to TLR4 activates autophagy through the MAPK/P38 (mitogen‐activated protein kinase) signaling axis, while the binding of lipoteichoic acid to TLR2 induces autophagy via the MAPK1/ERK2–MAPK3/ERK1 pathway.^259^, ^260^, ^261^ Thus, different TLR ligands exhibit variable autophagy‐inducing abilities.^262^, ^263^ Although autophagy levels were regulated by the severity of sepsis, mitochondrial biogenesis was successfully restored by injection of cell‐permeable TATI‐beclin‐1 through the PINK1/Parkin and AMP‐activated protein kinase (AMPK)/Unc‐51‐like protein kinase 1 signaling pathways and maintained sepsis cardiac function.^264^ Beclin‐1 plays a vital role in supporting PINK1/Parkin‐mediated mitophagy by interacting with Parkin. This interaction helps localize mitochondrial‐related membranes, leading to the tethering of ER mitochondria and the formation of mitochondrial autophagosome precursors.^233^, ^265^ The recruitment and activation of Parkin by the PINK1 protein on the mitochondrial outer membrane is the initial step in this process. Parkin then constructs ubiquitin chains on damaged mitochondria, facilitating their binding with LC3 on autophagosomes to induce mitophagy (Figure 1).^227^, ^266^, ^267^ Furthermore, crosstalk can occur between autophagy and apoptosis.^268^, ^269^ In a CLP mouse model of sepsis, T cell apoptosis was induced, while autophagy in CD4^+^ spleen cells and CD8^+^ spleen cells was downregulated.^3^, ^270^ In addition, mice with lymphocyte‐specific deletion of the Atg5 or Atg7 genes, which are needed for autophagy, had increased mortality, immune dysfunction, and T‐cell apoptosis compared with control mice. For example, T cell‐specific deletion of Atg5 in mice leads to increased Il‐10 production by T cells after CLP.^271^, ^272^ These findings suggest that inadequate autophagy results in sepsis‐induced immunosuppression.

## 3 POTENTIAL THERAPEUTIC TARGETS: SIGNALING MODULATORS

Due to sepsis being a complex disease condition, multiple targets and target external environment interactions are necessary for the occurrence and clinical manifestations of sepsis. Despite substantial improvements in care delivery, intensive drug therapy and immunosuppression continue to lead to multidrug‐resistant organisms and opportunistic infections, as well as long‐term outcomes such as organ failure, immunosuppression, and disability. Various candidate target studies have identified polymorphisms, but these results have not been consistently confirmed. To further understand the pathogenesis of sepsis and the results of new interventions, nine targets, such as GSDMD, HMGB1, and STING, were evaluated according to the important molecular signaling pathways of sepsis, which play vital regulatory roles in the occurrence and development of sepsis.

The nine crucial targets in dangerous liaisons act as linchpins in the events that lead to sepsis. One of the signature events in mediating protective innate immune responses against invading pathogens and microbial infections is the cleavage and pore formation of GSDMD. This event plays a decisive role in regulating inflammation, pyroptosis, and coagulation.^273^ Regarding the coagulation process, extracellular HMGB1 has been shown to enhance TF procoagulant activity by promoting the externalization of PS to the outer plasma membrane. Intracellular HMGB1 binds to bacterial endotoxin and transmits it to the cytoplasm through the RAGE receptor on vascular ECs and macrophages.^274^ This transmission triggers caspase‐11‐mediated pyroptosis, eventually resulting in shock, multiorgan failure, and death. Although CASP‐11 activation converges as an upstream signal for GSDMD‐dependent PS exposure, other mechanisms, such as NETosis, platelet activation, and disruption of the endothelial Tie2 axis, may contribute to the coagulation cascade. STING, a classical innate immune sensor that stimulates the expression of IFN in response to DAMP (cytoplasmic DNA), has been identified to be involved in coagulation by triggering ER stress‐induced activation of GSDMD.^275^, ^276^ STING interacts with the major calcium channel ITPR1 to promote ER calcium efflux, which is necessary for CASP‐1/11/8‐induced cleavage and activation of GSDMD. Eventually, ER stress‐mediated pore formation in GSDMD triggers pyroptosis and subsequent release of TF. Persistent innate immune activation by STING continuously promotes the process of inflammasome coagulation present in GSDMD pores.^277^, ^278^ SQSTM1 is released into the extracellular environment by macrophages through two mechanisms (active secretion or passive release). Extracellular LPS induces SQSTM1 lysosomal secretion through activation of the TLR4–MYD88–STING1 pathway, while intracellular LPS triggers the release of SQSTM1 through pyroptosis via activation of the CASP11–GSDMD–GSDMDN pathway.^279^ After release, SQSTM1 binds to the receptor insulin receptor (INSR) to activate the NF‐κB pathway, leading to the polarization of proinflammatory macrophages and ultimately mediating septic death in mice through hyperinflammation and coagulation.^280^ Recent studies have shown the important role of regulating immune checkpoints in sepsis‐induced immunosuppression. HMGB1 is secreted by melanocytes, which in turn activates the RAGE and promotes NF‐κB and IRF3‐dependent PD‐L1 transcription in melanocytes. In addition, PD‐1 and PD‐L1 are important regulators that inhibit T cell receptor‐induced activation signals, and regulation of the PD‐1/PD‐L1 axis can correct the immunosuppression of sepsis.^281^, ^282^, ^283^ Oxidative stress is caused by an imbalance between the production of ROS and the body's ability to remove these toxic intermediates. Park 7 is an unparalleled antioxidant that independently functions in cellular defense against ROS, without relying on other antioxidant pathways. It plays a vital role in regulating the generation of ROS through its interaction with p47phox, a subunit of NADPH oxidase.^284^, ^285^, ^286^ ROS act as a pivotal mediator, initiating the TLR signaling pathway to activate macrophages. The mounting evidence strongly suggests that Park 7 acts as an antagonist of sepsis‐induced immunosuppression, indicating its potential as a novel therapeutic target for reversing impaired immunity caused by sepsis. The activation of complement leads to the discharge of C3a and C5a, which possess immense proinflammatory properties, such as the recruitment and activation of leukocytes, ECs, and platelets. While complement activation plays a crucial role in the initial defense mechanisms of the immune system, uncontrolled activation leads to septic shock.^86^ SESN2, a stress‐inducible protein, inhibits the long‐lasting activation of the NLRP3 inflammasome by inducing mitophagy in macrophages to eliminate damaged mitochondria. SESN2 performs a dual function in the induction of mitophagy activated by inflammasomes. First, SESN2 initiates “mitochondrial priming” by marking mitochondria for recognition by the autophagy machinery. To prepare the mitochondria for this process, SESN2 causes the aggregation of mitochondria near the nucleus, mediated by the aggregation of SQSTM1 and its binding to the ubiquitin linked to lysine 63 (Lys63) on the mitochondrial surface. Second, SESN2 stimulates specific autophagy mechanisms to breakdown the targeted mitochondria by increasing the levels of ULK1 (UNC‐51‐like kinase 1) protein.^287^ Furthermore, prolonged LPS stimulation induces an increase in SESN2 expression in macrophages by mediating NO (nitric oxide) through NOS2 (nitric oxide synthase 2, inducible).^288^ Therefore, mice deficient in SESN2 display impaired mitophagy in two distinct sepsis models, leading to hyperactivation of the inflammasome and ultimately elevated mortality. Targeting JAKs and/or STATs could become an important approach to reducing mortality in septic shock, as these proteins are clearly associated with immune dysfunction and MOF. Original JAK–STAT‐based therapies should be designed to treat sepsis‐induced immunosuppression and may reach novel targets in the field.^289^, ^290^ Furthermore, phosphorylated STAT may provide creative therapeutic markers to drive such therapy. Therefore, we selected these nine targets for a detailed description of their major regulatory molecular pathways and potential in the development of sepsis.

### 3.1 GSDMD: promoters of inflammation and pyroptosis

Recent studies have found that gasdermin (GSDMs) can induce the transition from apoptosis to pyroptosis, and induce inflammatory and antitumor immunity. Activation of GSDMs can occur through caspases and granzymes, resulting in apoptosis in various scenarios.^150^, ^151^, ^291^, ^292^ For instance, when the level of GSDM expression is insufficient to induce pyroptosis, caspase/granzyme‐driven apoptosis can be converted into pyroptosis by expressing GSDMs.^293^, ^294^, ^295^ The occurrence of pyroptosis is primarily associated with inflammation, which arises from the assembly of a multiprotein complex in response to PAMPs, DAMPs, or environmental stress. Notably, the regulatory network of GSDMD, which plays a significant role in inflammatory diseases, has demonstrated its ability to safeguard animals against septic shock or lethal endotoxemia.^296^, ^297^ Hence, GSDMD is regarded as a promising target for sepsis treatment.

The pore‐forming properties of the gasdermin family play a crucial role in pyroptosis, which is a form of cell death with inflammation.^298^ When active caspase‐1/4/5/11 cleaves GSDMD (gasdermin protein), it releases the functional gasdermin‐N domains. These domains have the ability to induce liposome permeation and form intrinsic pores in the cell membrane. This process ultimately leads to the formation of membrane pores and the release of inflammatory cytokines, specifically IL‐18 and IL‐1β. Studies have shown that deleting GSDMD can block pyroptosis and provide protection against sepsis, systemic blood coagulation, and multiorgan damage.^279^, ^299^, ^300^ GSDMD not only induces cell death but also contributes to necro‐inflammation by releasing TF in macrophages and ECs, resulting in significant tissue damage.^121^, ^301^ Moreover, evidence suggests that GSDMD is involved in coagulation responses during sepsis. It promotes the release of coagulation factor III (F3) into the bloodstream, leading to systemic coagulation. Activation of GSDMD through caspase 11 (CASP11) is needed for this process.^60^, ^302^ Interestingly, GSDMD‐independent pyroptosis relies on the activation of the purinergic receptor P2 × 7 (P2RX7), but disrupting P2RX7 does not affect the coagulation cascade in septic mice.^302^, ^303^ This emphasizes the unique role of GSDMD in promoting coagulation.^304^, ^305^ Additionally, experiments with intraperitoneal injections of nuclear sulfonamides, such as pseudokinase, have shown that they can directly inhibit GSDMD. This inhibition offers protection against sepsis in mice compared with control groups.^273^, ^304^ Based on these findings, the GSDMD system is considered a potential therapeutic target for treating sepsis and warrants further investigation.

### 3.2 HMGB1: crucial intermediators between inflammation and coagulation

HMGB1 was initially identified as a protein that binds to the nucleus and enhances the transcription of genes by ensuring the stability of nucleosome formation. Follow‐up investigations have validated the involvement of HMGB1 in various cellular processes, including DNA recombination, repair, replication, and gene transcription. These activities are facilitated by the presence of repeated positively charged domains (HMG boxes) within the N‐terminal region of HMGB1.^306^ Thus, human or mouse HMGB1 protein not only functions as a nuclear factor but also functions as a vital cytokine mediating responses to infection, injury, and inflammation.^307^ Once it is actively secreted by immune cells or the epithelium or released by damaged cells, extracellular HMGB1 can serve as a DAMP and lethal mediator in critical diseases such as sepsis and COVID‐19 in both mice and humans.^308^, ^309^ Recent research has indicated that HMGB1 may potentially trigger inflammasome activation through cell interactions in mouse sepsis models.^55^, ^310^ As a late‐phase alarmin in sepsis, HMGB1 interacts with RAGE to deliver cytosolic LPS, consequently activating the NLRP3 inflammasome in murine macrophages and lung ECs, leading to caspase‐11‐dependent pyroptosis.^274^, ^310^, ^311^ HMGB1 also binds to cellular receptors for RAGE that enter the lysosomes of macrophages and ECs and trigger activation of the transcription factors NF‐κB and MAPK.^312^ In addition, HMGB1 destabilizes lysosomal membranes and promotes LPS release into the cytoplasm, leading to pyroptosis, and thus promoting lethal coagulation.^313^, ^314^, ^315^ These results establish a pathway dependent on DAMP for the transportation of LPS into the cytoplasm, triggering the activation of CASP11 and coagulation. This pathway operates independently from the non‐DAMP pathways previously documented, which involve the release of outer membrane vesicles by bacteria or the involvement of human guanylate binding protein 1 (GBP1).^316^, ^317^, ^318^

Note that, although the expression of CASP11 induced by LPS requires TLR4, it is not necessary for sepsis death induced by poly(I:C), which may help explain the unsatisfactory results of clinical trials attempting to treat sepsis with TLR4 inhibitors.^121^, ^319^, ^320^ These findings suggest that in mice, the activation and formation of pores by HMGB1 are necessary for F3 activation through the exposure of phosphatidylserine (PS).^306^, ^313^ As a result, PS‐binding proteins such as lactadherin and MFG‐E8 can restrict cytoplasmic F3 activation induced by LPS in murine macrophages.^321^, ^322^ An unexpected discovery was that glycine, an osmoprotectant, inhibits the release of F3 from infected BMDMs but does not affect cytoplasmic F3 activation induced by LPS in murine macrophages.^302^, ^303^, ^323^, ^324^, ^325^ Several possible reasons exist for these seemingly contradictory outcomes: the disruption of the plasma membrane by Ninjurin 1 during lytic cell death, including pyroptosis, might also contribute to HMGB1 release and F3 activation during lethal infection in mice.^278^, ^324^, ^326^, ^327^ In vitro studies have concluded that GSDMD‐mediated influx of Ca^2+^ promotes F3 activation, partly through anoctamin 6 (TMEM16F).^328^, ^329^ In contrast to pyroptosis mediated by GSDMD‐induced release of HMGB1 in macrophages, GSDMD‐deficient mice typically release HMGB1 during endotoxemia.^243^, ^299^, ^330^, ^331^ Consequently, these findings suggest the possibility of overlapping and diverse functions of GSDMD and inflammasomes in regulating HMGB1 release, which may play a role in feedback mechanisms controlling inflammation and coagulation.

### 3.3 STING: multiple regulatory functions of STING in sepsis

Stimulator of interferon response, cGAMP interactor 1 (STING1 or TMEM173), is a transmembrane adaptor protein that is commonly found in the ER of both human and mouse cells.^332^ Recently, the activation of the STING1 pathway in myeloid cells, such as macrophages and monocytes, has been associated with inflammation, coagulation, and tissue damage in mouse models of sepsis. These models include polymicrobial infections or bacteremia induced by *Escherichia coli* or *Streptococcus pneumoniae*.^333^ To better understand the role of STING in regulating the innate immune response to sepsis, extensive research has been conducted to identify key regulators of the STING pathway.^276^, ^334^, ^335^ Among the potential targets, anapestic lymphoma receptor tyrosine kinase (ALK) has been proposed as a novel regulatory factor for the STING‐mediated innate response. ALK, a receptor tyrosine kinase commonly associated with tumors, exhibits relatively low expression levels in healthy individuals.^336^

The binding of ALK/EGFR has been theoretically demonstrated to activate STING in innate immune cells dependent on Akt. This finding provides supporting evidence for a novel signaling pathway implicated in the development of sepsis pathogenesis and septic shock.^337^ Therefore, the ALK–EGFR–Akt pathway plays a critical regulatory role in the STING‐mediated innate immune response, which has been proven to be another important pathogenesis of fatal sepsis. In previous studies, STING mutations were shown to perturb ER calcium homeostasis and drive T cells to overreact to ER stress induced by TCR signaling.^338^ This chronic increase in STING‐mediated ER stress can effectively trigger the death of T cells due to apoptosis.^339^ Additionally, the severity of DIC and mortality in sepsis patients is linked to the expression of STING and GSDMD.^279^, ^292^, ^303^ Systemic coagulation mediated by STING is not dependent on the classical STING‐induced pathway. It is noteworthy that during endotoxemia in mice, STING1‐induced apoptosis of CD4^+^ T cells promotes inflammation‐induced immunosuppression, indicating the potential of STING as a target for treating T cell‐mediated diseases.^278^, ^324^ Moreover, when cytosolic DNA is recognized by cyclic GMP–AMP synthase (CGAS), activated STING1 can be moved to lysosomes, causing lysosomal rupture and subsequent K^+^ efflux, ultimately leading to the activation of the NLRP3 inflammasome.^340^, ^341^ GSDMD has been shown to either limit or induce CGAS‐mediated STING1 activation by measuring the production of type I interferon genes in murine macrophages and ECs, respectively. This suggests that GSDMD has a dual function in the feedback control of STING1 activation.^277^, ^342^ In summary, the activity of STING1 is regulated by various binding proteins or posttranslational modifications associated with immunity, autophagy, and cell death, highlighting the complex contribution of STING1 to the development of diseases, particularly sepsis and coagulation‐related conditions.^275^, ^343^ Recently, the STING pathway has been proposed as another viable therapeutic target for sepsis due to its multiple effects on immune homeostasis, coagulation, and inflammation.

### 3.4 C3a/C5a: the double‐edged sword of the innate immune system

In the complement system, the most crucial molecule to inhibit is C3 because it obstructs all connections of the entire system to C3 and downstream.^71^, ^78^, ^344^, ^345^ Hence, there is a significant potential to decelerate all complement‐mediated functions by obstructing C3.^73^, ^346^ It was verified that a synthetic inhibitor of C3 convertase (Compstatin) not only impeded complement activation during *E. coli*‐induced sepsis in baboons but also mitigated other inflammatory responses, activation of coagulation, and multiple organ failure.^71^, ^347^ It is noteworthy that while blocking C3, the cascade is not completely intercepted by the other constituents, since activation of the classical and lectin pathways produces some C5 convertase, and some activation of the terminal pathway transpires.^130^, ^348^ Nevertheless, blocking C3a or C3aR might also serve as an alternative to inhibiting the anaphylactic toxin activity of C3 cleavage, although this approach has not been tested in clinical trials. C5a demonstrates the highest potency as an inflammatory mediator of the complement system.^69^, ^79^, ^349^ Blocking C5a signaling improved outcomes in multiple *E. coli*‐induced sepsis animal models, including monkeys, mice, and rats with a variety of types of microbial sepsis.^350^, ^351^ According to a recent investigation, baboon survival was enhanced, coagulopathy was decreased, and endothelial and barrier function were maintained in an *E. coli*‐induced sepsis model through the utilization of RA101295, a C5 peptide inhibitor. This study observed a noteworthy reduction in consecutive identical words, thereby demonstrating the effective role of RA101295 in combating sepsis‐related challenges.^71^, ^352^ Therefore, C3a and C5a receptors may have different regulatory functions in sepsis. Nevertheless, recent research has illustrated that the utilization of a C5a receptor blocker resulted in improved survival rates across various forms of microbial sepsis. Conversely, the administration of a C3a receptor blocker led to diminished survival rates.^353^, ^354^, ^355^ Consequently, it can be concluded that complement activation plays a significant role in the development of multiorgan dysfunction syndrome during sepsis. Consequently, the inhibition of complement fragments and/or their receptors could be a valuable approach in sepsis treatment. Despite the successful development of agents specifically designed to inhibit complement activation, their complexity and, in some instances, unfavorable effects hinder their therapeutic applicability. Further investigation into the alterations in complement activation throughout sepsis may aid in identifying patients and interventions that are most likely to yield positive outcomes.

### 3.5 Park 7: park7 is a unique antioxidant that blocks inflammation and ROS

Macrophages, as crucial cells in the innate immune system, have a significant impact on inflammation and immune processes.^356^, ^357^, ^358^ In the initial phases of sepsis, macrophages commonly exhibit proinflammatory characteristics. However, an excessive inflammatory response by macrophages can trigger macrophage apoptosis, disrupt macrophage polarization, and ultimately lead to immunosuppression.^359^, ^360^ ROS can activate various TLR signaling pathways, subsequently governing macrophage function. These ROS are generated through the activation of NADPH oxidase.^361^, ^362^ Currently, the indispensable role of Parkinson's disease protein 7 (Park 7) in regulating the production of ROS by interacting with P47PHOx, a subunit of NADPH oxidase, is well known.^362^, ^363^, ^364^ This interaction activates NADPH oxidase, leading to an increase in ROS levels in macrophages, thereby initiating TRL signaling and enhancing macrophage function to counteract sepsis‐induced immune suppression.^365^, ^366^ Consequently, mice with Park7 KO can serve as an ideal model for studying advanced sepsis.^367^, ^368^ More specifically, during the later stages of sepsis, the impaired activation of macrophages can be attributed to the dampened TLR/NF‐κB and/or TLR/MARKs signaling pathways induced by LPS/LTA/PGN/proinflammatory cytokines.^285^, ^286^, ^369^ P47phox, which serves as the proenzyme subunit of NADPH oxidase, plays a critical role in the assembly of NADPH oxidase.^370^, ^371^ The interaction between Park 7 and p47phox facilitates the phosphorylation and membrane translocation of p47phox, ultimately leading to the formation of the holoenzyme complex.^372^, ^373^ Subsequent activation of NADPH oxidase results in the generation of ROS, which, in turn, activate the MAPKs and NF‐κB signaling pathways downstream of TLR signaling, thereby promoting macrophage activation.^374^, ^375^, ^376^ Activated macrophages play a crucial role in preventing sepsis‐induced immune suppression by releasing proinflammatory cytokines, eliminating pathogens, polarizing toward the M1 phenotype, and enhancing autophagy capacity.^377^, ^378^, ^379^ Based on this knowledge, targeting the Park 7/P47PHOX/ROS axis could prove to be an effective therapeutic strategy against sepsis‐induced immunosuppression.

### 3.6 SQSTM1: ‘Jack of all trades’ in health and septic shock

Sequestosome 1 (SQSTM1/P62) serves not only as a receptor for macroautophagy/autophagy but also as a versatile protein engaged in signaling cascades during inflammation and oxidative stress.^380^, ^381^ In vitro and in vivo experiments have emphasized the role of extracellular SQSTM1 as a deadly inflammatory mediator leading to sepsis and septic shock mortality, according to recent research.^280^, ^382^ In brief, SQSTM1 release occurs during tissue damage or microbial invasion, mainly in two ways: passively and actively. Passive release of SQSTM1 from human or mouse macrophages and monocytes is induced by the activation of TLR4‐mediated transactivation of the Sqstm1 gene and STING1‐mediated phosphorylation of the SQSTM1 protein at Ser403 in the active mode.^383^, ^384^ In vitro, cytoplasmic LPS also triggers GSDMD‐dependent pyroptosis, which further facilitates the passive release of SQSTM1 from macrophages and monocytes.^279^, ^280^ Following its release, extracellular SQSTM1 binds to the INSR located on the membrane, resulting in the activation of glycolysis. This activation subsequently leads to the production of proinflammatory cytokines in a manner dependent on the transcription factor NF‐κB.^385^, ^386^ Functionally, the pathway involving SQSTM1 and INSR can have a significant impact on tissue damage, systemic inflammation, organ failure, and mortality in experimental sepsis mouse models when either genetically deleted or pharmaceutically inhibited.^387^, ^388^ Moreover, the severity of sepsis in patients has been found to be associated with the activation of the SQSTM1–INSR pathway.

The SQSTM1–INSR axis activates NFKB and triggers inflammation, which occurs in macrophages via a pathway involving phospholipase C gamma 1 (PLCG1)‐dependent lipid peroxidation. This process leads to elevated pyroptosis, a form of cell death.^389^, ^390^ Blocking SQSTM1 by administering neutralizing monoclonal antibodies or deleting the INSR or SQSTM1 genes in bone marrow cells (BMCs) effectively safeguards mice from fatal sepsis.^279^, ^280^ Patients with bacterial sepsis show increased levels of SQSTM1 and INSR mRNA in peripheral blood mononuclear cells, along with elevated serum SQSTM1 concentrations. This finding further supports the idea that the SQSTM1–INSR pathway may have a detrimental role in human sepsis.^391^, ^392^ Additionally, higher serum SQSTM1 concentrations have been found to be an independent risk factor for patients with steatosis and liver inflammation.^36,398^ Therefore, revealing its proinflammatory potential would help shed light on the regulatory role of extracellular SQSTM1 in the pathogenesis of various inflammatory diseases.

### 3.7 PD‐1/PD‐L1: regulatory immune checkpoints in sepsis

PD‐1/PD‐L1 signaling pathways play a crucial role in the immunosuppression caused by sepsis. These pathways act through both the innate and adaptive immune systems, leading to various detrimental effects. These effects include the depletion of T cells, lymphopenia, apoptosis, impaired proliferation, decreased production of proinflammatory and anti‐inflammatory factors, compromised secretion of antigen‐presenting cells, and reduced functionality of BMCs.^282^, ^393^ Specifically, PD‐1/PD‐L not only play critical roles in tumor‐infiltrating T lymphocytes but are also highly expressed in nonimmune cells, and the expression level is correlated with organ injury.^27^, ^394^ In addition to inhibiting the number and functional activity of T cells, PD‐1 can also bind to its receptor PD‐1 to deliver a coinhibitory signal to negatively regulate T cell activation and mediate apoptosis.^395^, ^396^, ^397^, ^398^ In short, PD‐1 binding to PD‐L inhibited T cell activation and cytokine production, which may provide new guidance for sepsis defense.^399^, ^400^

Enhanced PD‐L1 expression has been documented in various cell types in sepsis.^283^, ^400^, ^401^, ^402^, ^403^ During sepsis, PD‐L1 expression is increased on both stromal cells and DCs.^404^, ^405^ Splenic ECs obtained from sepsis patients exhibited higher levels of PD‐L1 expression compared with the spleens obtained from patients who experienced brain death or needed emergent splenectomy due to trauma.^401^, ^402^, ^406^, ^407^, ^408^, ^409^ However, multiple other studies have demonstrated a higher expression of PD‐1 in memory subsets of B cells and CD4^+^ T cells among individuals suffering from sepsis.^410^, ^411^ Experimental investigations conducted on septic mice have confirmed the reprogramming of monocytes or macrophages during sepsis, resulting in decreased expression of human leukocyte antigen‐DR (HLA‐DR) and the release of proinflammatory cytokines. This is then followed by a decline in antigen presentation capability and phagocytosis.^33^, ^412^, ^413^, ^414^ Elevated levels of PD‐1/PD‐L1 observed on the surface of peripheral blood monocytes or macrophages have been associated with reduced phagocytosis, a decrease in the secretion of proinflammatory cytokines such as IL‐6 and TNF‐ɑ, and an increase in the release of anti‐inflammatory cytokines such as IL‐10.^398^, ^415^ Furthermore, clinical trials have revealed a decrease in the expression of HLA‐DR and CD86, which are costimulatory molecules, on monocytes/macrophages in sepsis patients. Conversely, the expression of PD‐1 and PD‐L1 is elevated, indicating a correlation with cellular dysfunction. These biomarkers may serve as indicators of monocyte or macrophage dysfunction in patients with sepsis.^408^ In vitro studies have shown that PD‐1‐mediated immune dysfunction of macrophages during sepsis occurs through the alteration of macrophage migratory ability. However, the administration of an anti‐PD‐1/PD‐L1 antibody can reverse these dysfunctions in sepsis patients.^416^, ^417^ Similarly, mice deficient in PD‐1 have demonstrated an enhanced phagocytic capacity in macrophages. Kupffer cells, which act as macrophages in the liver and remove bacteria from the bloodstream, also exhibit increased cytokine production during sepsis.^403^, ^417^, ^418^, ^419^, ^420^ The results presented here reinforce the concept that the abnormal activation of the PD‐1/PD‐L1 pathway is the main reason behind immunotherapy in individuals suffering from sepsis.

### 3.8 JAK/STAT: JAK–STAT signaling blocks sepsis‐induced immunosuppression

Janus kinases and signal transducers and activators of transcription (JAKs–STATs) are pivotal signaling components downstream of cytokine receptors.^289^ During the onset of sepsis, the two main causes of death are SIRS‐induced multiple organ failure and sepsis‐induced immune rejection responsible for late infections.^421^, ^422^ The signal conduction of these two lethal processes is involved in the regulation of JAK–STAT pathways.^423^ SIRS is mainly mediated by STAT1 and STAT4, whereas compensatory anti‐inflammatory response syndrome is predominantly mediated by STAT3 and STAT6, with the involvement of JAK1‐2 and tyrosine kinase (TYK) 2 for both cases.^424^, ^425^ Moreover, the JAK–STAT pathway also plays an essential role in cell proliferation and apoptosis, mainly mediating sepsis‐triggered emergency hematopoiesis and sepsis‐induced organ dysfunction.^290^, ^426^ Thus, targeting the JAK–STAT signaling pathway in sepsis may reduce sepsis‐induced MOF and sepsis‐induced immunosuppression. The recent successful development of JAK–STAT targeted therapy in oncology and hematology has provided new insights for the treatment of sepsis patients.

### 3.9 SESN2: the protector of the immune system

Sestrin2 (SESN2) is a highly evolutionarily conserved protein associated with cellular responses to various stresses; it protects against oxidative stress, DNA damage, hypoxia, nutritional stress, ER stress, autophagy, metabolism and inflammation.^110^, ^427^, ^428^ SESN2 functions as an antioxidant, activating AMPK, and suppressing mTORC1 signaling. Initially, discovered as a downstream mediator of p53, it is induced by various detrimental environmental strains, including oxidative stress, ER stress, energy stress, and age‐ and obesity‐related metabolic disorders.^17^, ^429^ SESN2, regulates autophagy, ER stress, and inflammasome activity under various conditions, exhibiting pleiotropic biological functions in cell homeostasis and metabolic homeostasis.^430^ Particularly, when facing ER stress, SESN2 is upregulated and exerts a protective effect against ER stress‐induced cell death. In situations of high stress, the activation of ER stress occurs as a result of the build‐up of proteins that are unfolded or improperly folded within the lumen of the ER. This activation is then followed by the initiation of three branches within the pathway for ER stress. SESN2 expression is upregulated by PKR‐like ER kinase (via eukaryotic translation initiation factor 2α and activating transcription factor 4), inositol‐requiring enzyme 1 (via Xbox binding protein 1, TRAF2 and c‐Jun N‐terminal kinase [JNK]) and activating transcription factor 6. SESN2 functions as a regulatory mechanism to mitigate the cellular response to ER stress or ER stress‐induced apoptosis.^24^ The results showed that hypoxia and NO strongly induced the expression of SESN2 in hypoxia‐induced factor‐1α, and the activation of SESN2 could prevent the peroxidation of peroxiredoxin, thus also acting as a cell protector.^287^ SESN2 expression has been detected in various immune cells, including monocytes, macrophages, natural killer (NK) cells, and T lymphocytes.^288^ Overexpression of SESN2 reduced ER stress‐related cell death, and SESN2 knockdown exacerbated the extent of ER stress, resulting in enhanced ER stress‐mediated apoptosis.^24^ SESN2 expression may exert beneficial effects on immune cell function by activating AMPK, inhibiting mTORC1 signaling, inhibiting JNK pathway activation, reducing the degree of ER stress, activating autophagy, or attenuating the NLRP3 inflammasome.^288^ Therefore, SESN2 has multiple regulatory effects and could be a promising therapeutic target and play a protective role in various inflammatory diseases, such as sepsis.

## 4 SEPSIS‐RELATED BIOLOGICS: PRECLINICAL STUDIES AND CLINICAL TRIALS

In recent years, a large number of emerging studies have provided increasing theoretical support for the process of pathological response to sepsis. Management interventions that are customary, such as the administration of fluids to restore proper hydration and the provision of vasopressors for hemodynamic support, have proven to be successful in the initial resuscitation of sepsis. These interventions bring about a noteworthy enhancement in the overall clinical results of patients.^47^ However, targeting the underlying causes that trigger disruptive manifestations remains unclear. Numerous clinical investigations have noted indications of immune cell activation and dysregulation of the host response in individuals experiencing severe sepsis. These findings align with multiple well‐known molecular pathways.^27^ Therefore, the development of new agents targeting molecular mechanisms and targets provides a strong experimental basis for further exploration of the treatment of sepsis. Here, we review novel molecular modulators targeting the main regulatory mechanisms of sepsis as well as therapeutic targets in the case of fatal infection, as shown in Table 1. Table 1 mainly summarizes the target proteins that this part of the regulator regulates when it exerts its function and the regulatory mechanism involved. In addition, the dosage of the modulator used, the time of publication, etc., are summarized.

**TABLE 1:** Potential inhibitors and their impact on sepsis in preclinical studies.

Therapeutic molecules	Target	Targeted mechanism	Usage	Model	Publication time	Sources
Glycyrrhizin	HMGB1	Inhibits HMGB1 release and activity	In vitro: 5–50 μM In vivo: 10–50 mg/kg	In vitro: BMDM, THP1, NR8383 In vivo: endotoxemia, CLP (mouse or rat)	2020, 2016, 2017	^284^, ^309^, ^434^
MCC950	NLRP3	Attenuates NLRP3 activation and inhibits HMGB1 release	In vitro: 0.1–10 μM In vivo: 10–50 mg/kg	In vitro: BMDM, HMDM In vivo: CLP (mouse or rat)	2018, 2016, 2019, 2020	^284^, ^310^, ^435^, ^436^
LDK‐378	ALK	Inhibits ALK‐STING pathway and NLRP3 inflammasome activation	In vitro: 1–10 μM In vivo: 20 mg/kg	In vitro: BMDM; THP1; RAW264.7; J774A.1 In vivo: endotoxemia, CLP (mouse)	2017,2018	^437^, ^438^
Ac‐FLTD‐CMK	CASP1/4/5/11	Inhibits GSDMD cleavage by caspases‐1, ‐4, ‐5, and ‐11	In vitro: 10 μM	In vitro: BMDM	2018	^439^
Z‐IETD‐FMK	CASP8	Inhibits CASP8 to decrease GSDMD‐N production	In vitro: 10 μM	In vitro: BMDM	2020	^303^
U73122	PLCG1	Inhibits GSDMD‐N to relieve pyroptosis	In vitro: 10 μM In vivo: 30 mg/kg	In vitro: BMDM In vivo: CLP (mouse)	2020, 2018	^303^, ^385^
H‐151	STING1	Covalently binds to STING at the transmembrane cysteine residue site 91 to inhibit STING1	In vitro: 2 μM In vivo: 750 nM/mice	In vitro: primary human or mouse macrophages In vivo: Trex1^−/−^ mice	2020, 2018	^280^, ^440^
YQ128	NLRP3	Inhibits the NLRP3 inflammasome and brain penetration	In vitro: 10–100 μM In vivo: 10–20 mg/kg	In vitro: BMDM, J774A.1 In vivo: endotoxemia (mouse)	2019	^441^
FPS‐ZM1	AGER	A high‐affinity AGER‐specific inhibitor	In vitro: 0.1–1 μM In vivo: 10 mg/kg, 75 μg/day	In vitro: BMDM In vivo: endotoxemia, Acinetobacter baumannii infection (mouse)	2019, 2017	^442^, ^443^
Zileuton	ALOX5	Inhibits ALOX5 to relieve lipid peroxidation	In vitro: 5 μM In vivo: 30 mg/kg	In vitro: BMDM In vivo: endotoxemia (mouse)	2019	^442^
Disulfiram	GSDMD	Inhibits pyroptosis by blocking gasdermin D pore formation	In vitro: 1–30 μM In vivo: 15–50 mg/kg	In vitro: BMDM, THP1 In vivo: endotoxemia (mouse)	2020	^444^
TUDCA	ER stress	F3 release was rescued by inhibiting ER stress	In vitro: 50 μM In vivo: 200 mg/kg	In vitro: THP1 In vivo: CLP (mouse)	2020	^303^
4PBA	ER stress	F3 release was rescued by inhibiting ER stress	In vitro: 1 mM In vivo: 20 mg/kg	In vitro: THP1 In vivo: CLP (mouse)	2020	^303^, ^445^
Ruxolitinib	JAK1, JAK2	Reduce NO production by interfering with the NF‐κB pathway	In vivo: 0.67 mg/kg	LPS‐induced sepsis model	2020	^445^
Tofacitinib	JAK1, JAK2, JAK3	Restrain differentiation of T‐cells by inhibiting JAK–STAT pathway	In vivo: 15 mg/kg/day	*Staphylococcus aureus*‐induced sepsis	2020	^446^
STC3141	NETs	Neutralization of extracellular histones and NETs to reverse organ damage caused by excessive immune responses	In vivo: 100 mg/kg In vivo: 50 mg/kg	LPS‐induced acute respiratory distress syndrome/CLP (rat)	2023	

**FIGURE 4:**
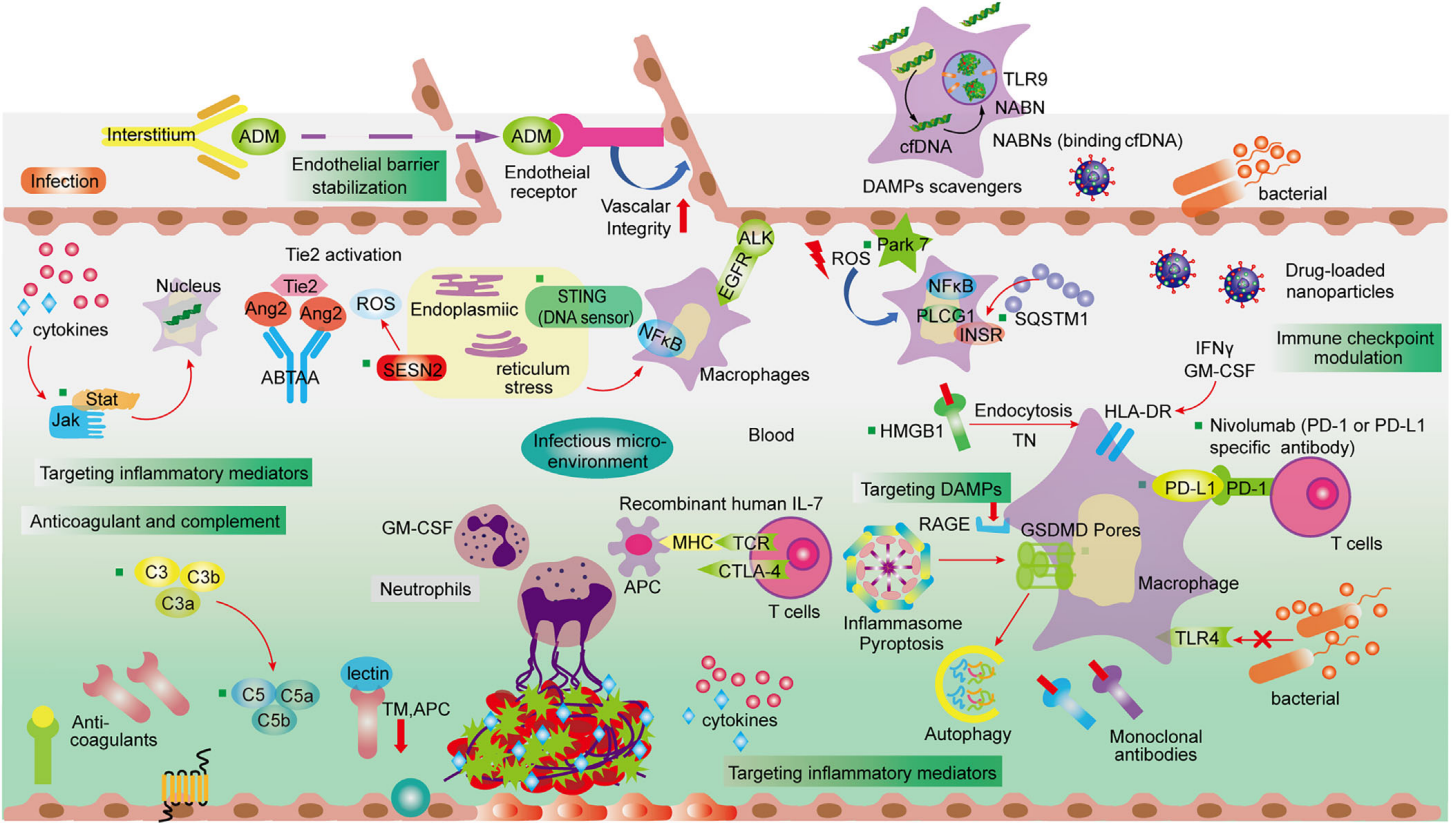
Potential therapeutic targets in sepsis intervention. Concerning the signaling pathways aimed at, the proposed therapeutic target for sepsis primarily encompasses the following pathway categories: (1) targeting DAMPs (including host cell stress), SESN2, and Park 7; (2) targeting inflammatory mediators (anti‐inflammatory), JAK–STAT, HMGB1, GSDMD, and SQSTM1; (3) immune checkpoint modulation, PD‐1/PD‐L1; (4) restoration of complement and anticoagulant properties, C3a/C5a; (5) endothelial barrier stabilization, Ang/Tie2; (6) targeting the endoplasmic reticulum, STING. The discovery of therapeutic targets is a promising new strategy that has a wide range of therapeutic effects in experimental studies of sepsis.

Several large phase II and III trials are currently underway (Table 2). Advances in supportive care have significantly improved the clinical outcomes of patients with sepsis, and new clinical trials are seeking further advantages over infusion, hemodynamic, and sedation therapies.^31^ However, due to differences in the clinical definition of sepsis and the multifaceted manifestations observed during the clinical course, the inclusion criteria for patients with sepsis and the observed clinical end points varied over the course of the trial.^431^ Some of the metrics of basic experiments do not seem to match the significance of clinical monitoring and cannot cover its impact on clinical efficacy, which is an inherent limitation of basic research. After the failure of therapeutic strategies for the inflammatory cascade in the early stage of sepsis development, the research focus of immunomodulatory therapy has shifted to enhancing the immune response in the late stage of immune paralysis. Recognizing that multiorgan failure is a major contributor to the clinical burden of systemic infections, early studies increasingly emphasized strategies to improve endothelial and epithelial–cell barrier function,^68^ biofunctionalism,^68^, ^432^ and active inflammation resolution pathways.^324^ The use of cell therapy, such as allogeneic stromal cells or mesenchymal stem cells, shows promise as a treatment option. These cells possess powerful functions in immunomodulation, antimicrobial activity, bioenergetics, and enhancement of barriers (Figure 4).

**TABLE 2:** Novel therapeutic molecules and their clinical impact on sepsis in clinical trials.

Therapeutic molecules	Mechanism	Population	Publication time	Study phase and design	Usage	Conclusion	Comment	Sources
Adrecizumab	ADM bounds to a non‐neutralizing antibody, interacts with receptors on endothelial cells and reduces vascular leakage and tissue oedema	300	2019	Phase II RCT	2 or 4 mg/kg	Adrecizumab represents an effective approach to the treatment of fatal syndrome	The patients with concentration of circulating bio‐ADM levels >70 pg/mL are recruited	
Human recombinant alkaline phosphatase	The inflammatory response was attenuated as a result of alkaline phosphatase dephosphorylating endotoxin and adenosine triphosphate	301	2018	RCT	1.6 mg/kg	In patients with severe sepsis‐associated acute kidney injury, the administration of recombinant human alkaline phosphatase did not result in improvement in renal function during the initial week of treatment	All patients were men	
Recombinant human IL‐7 (CYT107)	It reverses the dramatic loss of CD4^+^ and CD8^+^ immune effector cells	27	2018	RCT	10 μg/kg	CYT107 is a promising approach for treating sepsis by restoring adaptive immunity. These immune‐based therapies have the potential to provide comprehensive protection against various pathogens, with a specific focus on multidrug‐resistant bacteria in immunocompromised patients	**–**	
Nivolumab	The expression of PD‐1 and PD‐L1 is increased in patients with sepsis, which is associated with a decrease in T cells. Nivolumab reverses T cell exhaustion by blocking PD‐L1 to restore immune cell function, thereby improving immunosuppression	13	2020	Phase I/II study	480 or 960 mg	Over time, 480 mg and 960mg of nivolumab appeared to improve sepsis‐induced immune system injury	Japanese patients with immunosuppressive sepsis	^452^
Recombinant human thrombomodulin (ART‐123)	rhsTM binds circulating thrombin molecules and converts protein C to APC as an activation complex. And, rhsTM inhibits inflammation and organ damage caused by damage‐associated molecular patterns	800	2019	RCT	0.06 mg/kg/d	The 28‐day all‐cause mortality was not significantly reduced when administering a human recombinant thrombomodulin, as compared with the placebo	Patients with sepsis‐associated coagulopathy	
Reltecimod, AB103	Interaction of superantigens with dimers of the costimulatory receptor CD28 expressed on T cells mediates T helper type 1 cytokine responses	290	2020	Phase III RCT	0.5 mg/kg	In patients with severe NSTIs, early administration of reltecimod significantly improved the primary composite endpoint in the PP population	Patients ages ≥12 years with a surgical confirmation of NSTI, and organ dysfunction (mSOFA score≥3)
Ascorbic acid	–	167	2019	RCT	50 mg/kg	The 96‐h vitamin C infusion did not significantly improve organ dysfunction scores or change markers of inflammation and vascular damage compared with the placebo group	‐	
BMS‐936559	Restore or enhance T‐cell function	24	2019	Phase 1b	10–900 mg	BMS‐936559 exhibited good tolerability, no drug‐induced cytokine release, and immune status was restored within 28 days at high doses	Patients with sepsis, organ dysfunction, absolute lymphocyte count ≤1100 cells/μL	^456^, ^457^
STC3141				Phase II				
APAD				Phase I				

Recently, encouraging results have been observed in clinical randomized controlled trials (Table 2). Until then, monitoring a large number of targets and biomarkers related to innate immune responses, receptor expression, vascular barrier integrity, and tissue/organ damage can provide circumstantial evidence informing the extent of host response dysregulation, which is closely linked to the state of immune homeostasis associated with the clinical course and is critical for elucidating the pathophysiology of sepsis.^3^, ^433^ Therapeutic strategies aiming to remove and inhibit the respective molecular stimuli can be guided by biomarkers related to the innate immune response, including DAMPs, PAMPs, chemokines, and cytokines (Figure 4). These methods could potentially have a positive impact on promoting the early reversal of harmful occurrences. By employing alternative novel biological substances (nano‐sized particles filled with medications), the ongoing surveillance of immune cell activity levels can be utilized to detect individuals experiencing immune paralysis or abnormal proinflammatory host reactions. These measures provide the basis for future target‐specific guided interventions and personalized treatment.

Sepsis causes numerous immune response defects, leading to immune suppression and increased susceptibility to infection and death. In recent decades, great strides have been made in comprehending the mechanisms underlying the emergence of diverse pathogeneses associated with sepsis. Regrettably, these discoveries have not yet translated into efficacious therapies for bacterial sepsis. However, the mouse model of sepsis used, including injection of endotoxin, colonic ligation and perforation, and exogenous injection of pathogenic bacteria, does not fully mimic septic patients. Almost invariably, these models have been studied with the use of young, healthy, syngeneic animals, often without sophisticated monitoring, and with little or no supportive therapy (antibiotics, respiratory support including oxygen, fluid and vasopressor resuscitation, and renal replacement therapy). In addition to the significant differences in the composition and treatment of sepsis patients, the important species differences also lay an enormous potential for the inconsistency between preclinical and clinical trials in the development of drug release. To that end, many effective molecules (small molecule modulators and biologics), targets and related interventions for sepsis are being studied as hotspots (Figure 4). It will be imperative to further define the roles they play in experimental sepsis to facilitate the development of novel therapeutic approaches for clinical sepsis. Given that these inhibitors and targets have demonstrated the ability to impede disease progression in laboratory or animal models of sepsis, the anticipation for their assessment in human trials is pressing.

## 5 CONCLUSIONS AND PERSPECTIVES

The development of sepsis is characterized by hyperinflammation, immunosuppression, EC injury, DIC, multiple organ failure, and limited treatment options, which continues to be a formidable challenge for basic and clinical research. As of now, the attempts made to target only one mediator or target, such as IL‐1β or APC, have proven unsuccessful in diminishing mortality in sepsis patients. This distressing outcome warns us that the evolution of sepsis is a heterogeneous development process with multiple interfering factors and multiple regulatory pathways interacting. However, in recent years, basic research using cell experiments and mouse models has revealed that many key molecules exist in the complex regulatory network of organismal systems, and there are regulatory and regulated relationships between these key molecules, which play a decisive role in the development of the disease, thereby enhancing our comprehension of the essence, qualities, and outcomes of the sepsis response. Nonetheless, the significant genetic variations between humans and mice considerably restrict their utilization in sepsis patients. Despite this complexity, our objective is to enhance the understanding of the initial signaling, intermediate associations, final effectors, and feedback loops involved in sepsis. This will enable the identification of novel therapeutic targets and the development of treatments that can significantly benefit patients suffering from life‐threatening infections.

As noted previously, sepsis presents itself as a diverse syndrome that exhibits noteworthy distinctions in pathophysiology, clinical presentations, and consequences. The primary emphasis in many clinical trials examining innovative sepsis treatments has been on the preimmune status and pathophysiology exclusive to infection. These approaches generate high signal‐to‐noise ratios that do not result in favorable clinical outcomes. Past responses to sepsis‐induced homeostasis imbalances, such as complement, coagulation, mitochondrial damage, and endothelial dysfunction, have been improved. However, the history of clinical trials in sepsis has demonstrated that improvements in a single or a few physiologic markers in septic patients do not reverse clinical patient mortality, and the results do not necessarily translate into the clinical setting. Nevertheless, the primary hurdle lies in the fact that nearly every immune mediator assumes varied and interconnected functions in the pathophysiology of sepsis. This implies that the interception of these mediators can potentially disrupt the balance of homeostasis and accentuate disease‐amplifying networks. As a result, most clinical trials generally do not improve overall outcomes. A number of explanations have been proposed, including underdosing of interventional agents due to rapid clearance or rapid metabolic inactivation. The discovery of novel therapeutic targets targeting vital signaling pathways offers new hope for the treatment of sepsis, but a thoughtful preclinical approach will be essential. Effective interventions can be found to limit organ failure as quickly and effectively as possible in the treatment of sepsis. The complexity of sepsis‐associated organ dysfunction poses a challenge in achieving this goal, but the possibility of recovery underscores the need for identifying and implementing efficient measures.

In the past few years, studies examining the imbalance of inflammation, dysfunction in the immune system, damage to mitochondria, dysfunction in blood clotting, abnormalities in the network between the nervous and endocrine systems, stress in the ER, autophagy, and other mechanisms have greatly enhanced our understanding of how sepsis develops and uncovered potential targets for treatment as well as markers for prognosis. These findings hold promise for the prevention, diagnosis, and management of sepsis. Given the complexity of host responses and the diversity of pathophysiological pathways involved in sepsis patients, current “one‐target” and “one‐size‐fits‐all” approaches are unlikely to succeed. Since sepsis is a systemic disease involving multiple organs, patients currently die upon admission. However, clinical guidelines have implemented efficient management strategies to promptly control the spread of the source and ensure adequate cell perfusion through early and appropriate administration of antimicrobial therapy. Despite being costly and time consuming, this approach exhibits limited sensitivity and selectivity due to its inherent limitations. Therefore, there is an urgent need to develop alternatives to sepsis diagnostic systems. Currently, there is an absence of both a United States Food and Drug Administration‐approved medication and efficient, speedy tools for diagnosing sepsis in clinical settings. The direction that sepsis diagnosis is heading toward involves early detection and risk evaluation, based on the clinical traits and biomarkers of patients. Furthermore, more targeted investigations are needed to enhance our comprehension of the fundamental pathophysiology underlying sepsis mortality. Categorizing sepsis into distinct groups in the future might aid in identifying superior and more potent treatments, surpassing the efficacy of current approaches employed for sepsis management.

## AUTHOR CONTRIBUTION

Wendan Zhang wrote the paper. Honghong Jiang made the figures. Gaosong Wu, Pengli Huang, and Haonan Wang made the tables. Huazhang An revised the article. Weidong Zhang performed the supervision, project administration, and funding acquisition. Sanhong Liu contributed with the project administration, review, and editing. All authors have read and approved the final manuscript.

## CONFLICT OF INTEREST STATEMENT

The authors declare no conflicts of interest.

## ETHICS STATEMENT

Not applicable.

## Data Availability

Not applicable.
